# Supplementary figures and images for: Diversity and structural differences of bacterial microbial communities in rhizocompartments of desert leguminous plants
Source: PLoS One. 2020 Dec 22;15(12):e0241057. doi: 10.1371/journal.pone.0241057 (PMC7755220; doi:10.1371/journal.pone.0241057)

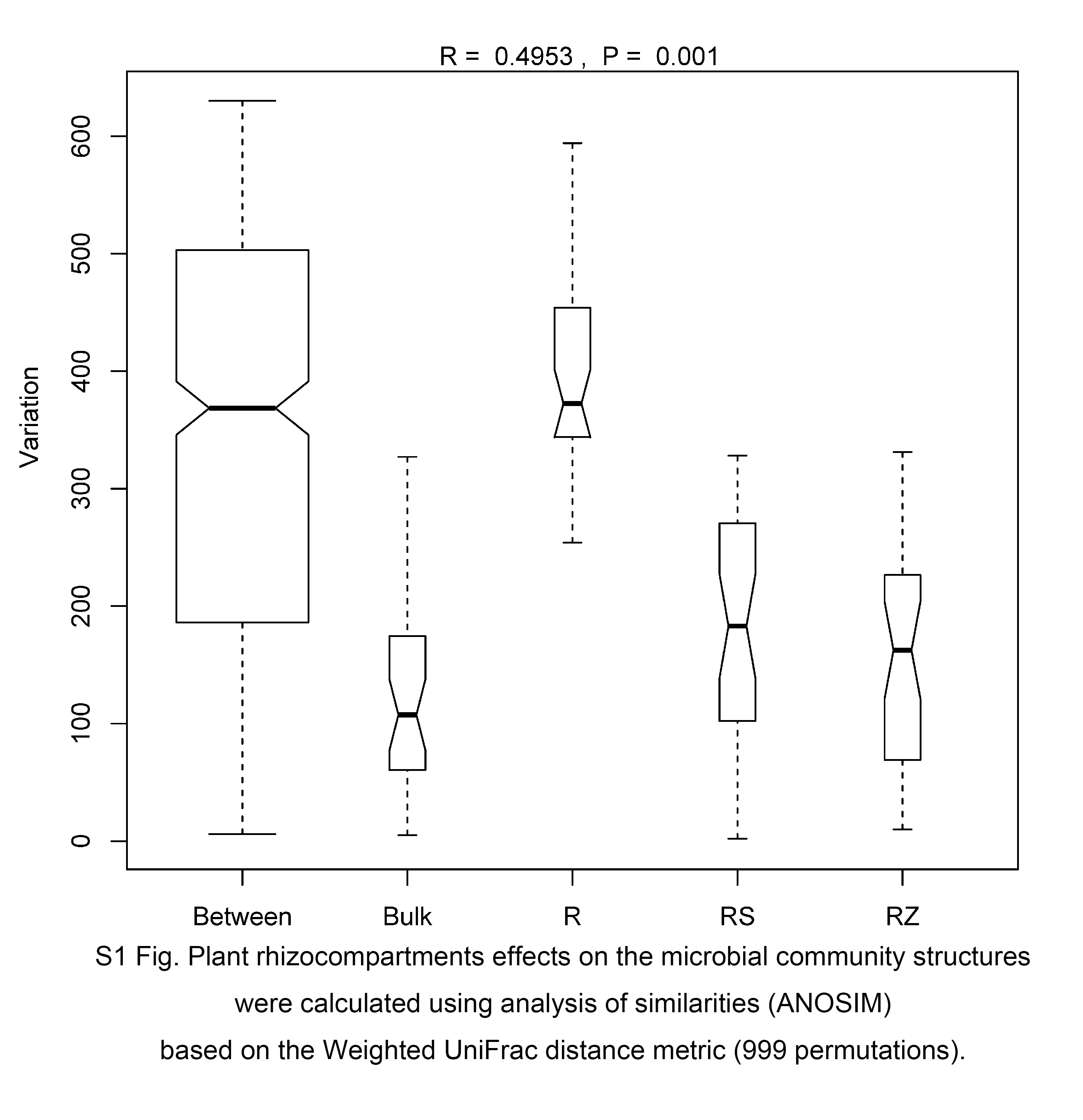

Supplement: S1 Fig — (TIF) [file pone.0241057.s001.tif]
