# Supplementary material for: Diversity and structural differences of bacterial microbial communities in rhizocompartments of desert leguminous plants
Source: PLoS One. 2020 Dec 22;15(12):e0241057. doi: 10.1371/journal.pone.0241057 (PMC7755220; doi:10.1371/journal.pone.0241057)
Supplement: Attachment — Submitted filename: rebuttal letter.docx [file pone.0241057.s005.docx]

**Rebuttal Letter**

22 February 2020

Dear Editor,

Re: Resubmission of PONE-D-20-03936

We had submitted a research article entitled ‘Diversity and Structural Variability of Bacterial Microbial Communities in the Rhizocompartments of Desert Leguminous Plants’ for publication in *PLOS ONE*.

However, in the process of review, Editor Zsofia Freund complied with the submission guideline of *PLOS ONE*, assumed that the submitted manuscript might be a resubmission of the manuscript (PONE-D-19-25078) previously reviewed and rejected by the journal. Therefore, the editor requested the authors to resubmit the manuscript in addition to give a point-by-point response to the reviewers’ comments and to summarize the revisions on the basis of their comments. I completely respect the submission guidelines of the journal as well as thank the reviewers for their generous comments on the manuscript. I have edited the manuscript to address the reviewers’ and editor’s concerns.

First, these two manuscripts submitted to the journal have totally different analysis thoughts, but both focused on the interaction between desert leguminous plants and the microbial community in the root system and the ecological niche differentiation in the rhizocompartment. The sampling strategy of the entire study was based on the growth stages of leguminous plants. Further, the bacterial community dynamics in the rhizocompartment was explored at different growth stages of the leguminous plants in the desert environment. The earlier manuscript (PONE-D-19-25078) includes data of the plant florescence (June) and the current manuscript includes data of the ripening period (September, 2018). In addition to the common, local and dominant xerophytic leguminous shrub species *Hedyssarum mongolicum* and *Hedysarum scoparium* (used in both the studies), this paper used a new, local and dominant leguminous species *Caragana microphylla* also to increase the sample size and to verify the earlier findings.

In the current manuscript, the suggestions and comments of reviewers were referenced in the setting of rhizocompartment, and the setting and analysis of the rhizocompartment types (i.e. nodule, root, rhizosphere soil, and root zone soil) were discarded because the difference in bacterial communities of nodule and root was not significant except for specific root endophyte microbiomes. The current manuscript focused on the setting of rhizocompartment types (i.e., roots, rhizosphere soil, root zone soil, and inter-shrub bulk soil) and is concerned more with the differentiation of soil microbial community structure under the shrub in the desert environment. We also focused on Specific core bacterial microbiomes which led to significant difference of structure composition between the rhizocompartments during niche differentiation process of rhizocompartment. Our findings provide data support for the interaction between specific plants and specific bacterial community in soil and offered a microbe-associated reference for evaluating the restoration of desert vegetation.

Since these two manuscripts are on the same subject (desert leguminous plant) and have similar themes, parts of methods used in sampling and sequencing analysis operation are the same, which make the description of parts of materials and methods section similar. In result and analysis section, Alpha diversity, Beta diversity, and species composition are basic contents which need description. Therefore, on the basis of combining the reviewers’ comments of the previously rejected manuscript, we have given a point-by-point response to the comments and suggestions of the reviewers from perspectives of two parts, materials and methods as well as results and analysis, and the revisions made are summarized as follows:

**Reviewer #1:**

1. **Did you get 5 shrub samples or one combined sample mixed from 5 shrubs in a quadrat?**

Response: Each plant species was sampled with three sample plots (100×100 m, with a spacing of 100 m). For each shrub species, five shrubs were randomly collected from each sample plot to collect root and soil samples. (Line 122-124) We followed the method described by Sun et al. (2017) to mix samples for the analysis of bacterial community diversity, each collected plant has three replicates according to its three sampling plots. The samples from the rhizocompartments of the five shrubs in the same sample plot were mixed, and these mixed samples were established according to the four rhizocompartment types of each plant species. (Lines 146–149)

2. **Why did you selected the V5-V7 regions of 16S rRNA in the study, but not V3-V4 regions which were reported to have the longest of variable region with good primer binding specificity, and selected by most studies.**

Response: Studies have shown that the selection of hypervariable regions V5–V7 from root tissues for sequencing effectively reduced host contamination (Zgadzaj et al. 2016; Liu et al. 2016; Sánchez-López et al. 2018）. (Lines 180-182)

**Reviewer #2:**

1. **Citation for climate data?**

Response: References have been added in Line 108.

2. **Citations or methods for texture/bulk density determination.**

Response: References have been added in Line 109.

3. **I don’t see a “quadrat method” in Bulgarelli et al. – I feel you’re referring to their extraction protocol? If so, I don’t feel comfortable with you saying you followed their root extraction protocol when you really did not – this is a major concern for me regarding this paper. For example, they define “rhizosphere” as soil clinging to roots – and they used THAT soil in their analysis, after washing it from the root surface. That’s not at all what you did, correct? You defined “rhizosphere” as soil within 1 cm of roots. From what I understand, most of your “rhizosphere” soil was NOT that clinging to the roots, but clinging soil was used only when needed to collect enough sample. I’m also confused about the large soil aggregates as being root zone soil – what’s the difference between that and your definition of rhizosphere soil? Wouldn’t these aggregates also fall, at least partially, within the 1 cm distance of the root that you define as the rhizosphere? Furthermore, there is no mention of attempts to keep samples sterile or avoid cross-contamination between samples. It’s hard to be sure of sample integrity without a discussion of that.**

Response: We are grateful to the reviewers for their time and detailed comments. We have tried our best to overcome the shortcomings in the methods of the rejected manuscript (PONE-D-19-25078). The authors have overcome the shortcomings of previous work, carefully treated the samples based on a brand new test design and the operation procedures proposed by references, and wrote the current version of the sampling strategy and the description of the sample processing parts in accordance with the actual operation process. The sampling and post-processing were done as described by Beckers et al. (2017) and Xiao et al. (2017). The soil attached to the rhizosphere was used only to analyze the soil physical and chemical properties (Line 129-130), while the soil obtained after root flushing was used for follow-up analysis of molecular biological. Post-processing details are in Lines 134–154 of this manuscript.

4. **Totally unclear as to how you designed your experiment and replicates. By “five samples of the same plant species under the four rhizocompartment types were mixed separately to prepare a total of 24 mixed samples”, I have no idea what you’re doing. What is being mixed? And how are the replicates created? Did you mix everything together and then separate them into three reps? Please clarify – same with the part 1 samples and part 2 samples. Also not sure what you mean by mixed soil sample.**

Response: Considering the reviewers' comments (PONE-D-19-25078) that the description was imperfect and difficult to be understood, we have modified the description to make it clearer and more concise. The modified description is as follows:

(i) Sampling strategy (Line 122-124)：Each species was sampled with three sample plots (100×100 m, with a spacing of 100 m). For each shrub species, five shrubs were randomly collected from each sample plot to collect root and soil samples.

(ii) Sample processing (Lines 145–154): In the subsequent sample preparation, according to the sample mixing processing method described by Sun et al. (2017) in the analysis of bacterial community diversity, each collected plant has three replicates according to its three sampling plots. The samples from the rhizocompartments of the five shrubs in the same sample plot were mixed, and these mixed samples were established according to the four rhizocompartment types of each plant species. Each mixed soil sample was homogenized and filtered through a 2 mm soil sieve to remove gravels and impurities. Each mixed sample was divided into two subsamples: Part 1 contained 36 DNA samples (3 shrub species × 4 rhizocompartment types × 3 replicates), which were stored at -80°C for molecular biological analysis; Part 2 contained 27 soil samples (3 shrub species × 3 rhizocompartment types × 3 replicates), which were air-dried for physicochemical analysis.

We believe that the above description about the two procedures would clearly indicate the experimental setting and processing methods.

5. **Citation for methods and differentiating TP from AP.** **Citation for sulphate method.**

Response: The International standard method published by the Institute of Soil Sciences, Chinese Academy of Sciences (1978) was adopted to measure AP, TP, NH^4+^-N, and NO^3-^-N indicators. (Line 159-164)

6. **When in the process was SWC measured? Before, during, after extraction? How long was soil exposed prior to measurement?**

Response: When the rhizocompartment was dug and sampled, the SWC was rapidly measured prior to the collection of corresponding samples. (Line 164-168)

7. **Citation for Mothur.**

Response: References have been added in Line 202.

8. **Results: Overall, the statistics are very poorly interpreted – often completely incorrect – and generalizations are made that are just not true. For example, 210-211 says, “a slight deficiency in NH_4_-N was observed in rhizosphere soils under both shrub species”. This is not true – NH4-N was only statistically different in the HM, not in both. Another example: Line 212-214 say that values under one shrub species were different than under the other – you cannot say that when you aren’t actually comparing them statistically. They may look different, but unless you run the appropriate tests and make statistically significant comparisons, you can’t say they’re different. Again, lines 214-216 claim that all nutrients except TP in rhizo and root zone soil are different in HS. That is not true – there are no significant differences between NH4+ levels in HS. It’s these simple oversights that make me doubt the integrity of your statistical interpretations. More examples follow, further below.**

**This is an example of misinterpretation that happens throughout the paper – when two values are the same, statistically, you cannot claim that one is greater or lesser than the other. You say that the Chao1 index, for example, has a significant hierarichical trend for both species – nodule < root < rz < rs, but this is not a correct interpretation of the results. For HM, the nodules and roots are EQUAL (both b) and the rs and rz are EQUAL (both a). For HS, rs and rz are EQUAL (both a). Nearly all of your hierarchical trends have this pattern to some extent – you can talk about possible trends, but you cannot claim that there is a significant difference or significant trend when the values are statistically equal.**

Response: Contents which basically needs description such as alpha diversity, beta diversity, and species composition of the rhizocompartment bacterial community were analyzed in both the studies. In combination with the comments of the previous reviewers, the authors have learned a lesson from the analysis of the results in the manuscript, and carefully checked the significance trend analysis involved in this manuscript, so as to prevent the occurrence of low-level errors that are similar to previous ones. These verified significant trend analysis run through the analysis of the results of each part in this manuscript. Thanks to the reviewers for the useful and detailed comments.

9. **Results: You add OTUs but not phyla or genera? Doesn’t make sense.**

Response: This suggestion proposed by the reviewers was very useful. We analyzed the top ten relatively abundant dominant bacterial genera corresponding to the enriched and depleted OTUs, and demonstrated in accordance with this recommendation, as shown in Figure 5.

Other comments by the previous reviewers are related to the discussion and conclusion of the rejected manuscript (PONE-D-19-25078) and will not be repeated here. However, the deficiencies in the rejected manuscript (PONE-D-19-25078) have been clearly addressed and referenced in this manuscript.

**Referencess**

1. Sun Y, Zhang YQ, Feng W, Qin SG, Liu Z, Bai Y, Yan R, Fa KY, 2017. Effects of xeric shrubs on soil microbial communities in a desert in northern China. Plant Soil. 2017; 414: 281–294. <https://doi.org/10.1007/s11104-016-3111-y>
2. Zgadzaj R, Garrido-Oter R, Jensen DB, Koprivova A, Schulze-Lefert P, Radutoiu S. Root nodule symbiosis in Lotus japonicus drives the establishment of distinctive rhizosphere, root, and nodule bacterial communities. Proc Natl Acad Sci U S A. 2016; 6, 113(49): E7996-E8005. https://doi.org/10.1073/pnas.1616564113
3. Liu H, Carvalhais L, Schenk P, et al. Effects of jasmonic acid signalling on the wheat microbiome differ between body sites. Sci Rep. 2017; 7: 41766. https://doi.org/10.1038/srep41766
4. Sánchez-López AS, Thijs S, Beckers B et al. Community structure and diversity of endophytic bacteria in seeds of three consecutive generations of Crotalaria pumila growing on metal mine residues. Plant and Soil. 2018; 422(1–2): 51–66. <https://doi.org/10.1007/s11104-017-3176-2>
5. Yu MH, Ding GD, Gao GL, Sun BP, Zhao YY, Wan L, Wang DY, Gui ZY. How the plant temperature links to the air temperature in the desert plant *Artemisia ordosica*. PLOS ONE. 2015; 10(8): e0135452-. <https://doi.org/10.1371/journal.pone.0135452>
6. Lai ZR, Zhang YQ, Liu JB, Wu B, Qin SG, Fa KY. Fine-root distribution, production, decomposition, and effect on soil organic carbon of three revegetation shrub species in northwest China. Forest Ecol Manag. 2016; 359: 381–388. <https://doi.org/10.1016/j.foreco.2015.04.025>
7. Beckers B, Op De Beeck M, Weyens N, Van Acker R, Van Montagu M, Boerjan W, Vangronsveld J. Lignin engineering in field-grown poplar trees affects the endosphere bacterial microbiome. Proc Natl Acad Sci U S A. 2016; 113 (8): 2312-2317. <https://doi.org/10.1073/pnas.1523264113>
8. Xiao X, Fan MC, Wang ET, Chen WM, Wei GH. Interactions of plant growth-promoting rhizobacteria and soil factors in two leguminous plants. Appl Microbiol Biotechnol. 2017; 101: 8485–97. <https://doi.org/10.1007/s00253-017-8550-8>
9. Kozich JJ, Westcott SL, Baxter NT, Highlander SK, Schloss PD. Development of a dual-index sequencing strategy and curation pipeline for analyzing amplicon sequence data on the MiSeq Illumina sequencing platform. Appl Environ Microb. 2013; 79: 5112–5120. <https://doi.org/10.1128/AEM.01043-13>

Thanks again to the reviewers and editors for the valuable comments, which helped improve our manuscript. We have carefully corrected the problems and deficiencies of the rejected manuscript (PONE-D-19-25078) which have been demonstrated in this manuscript. We hope that this manuscript is now suitable for publication in *PLOS ONE*.

On behalf of all authors.

Sincerely,

Ziyuan Zhou

E-mail: [zhou.zi.yuan@hotmail.com](mailto:zhou.zi.yuan@hotmail.com)

School of Soil and Water Conservation, Beijing Forestry University

No. 35, Qinghua East Road, Haidian District, Beijing 100083, P. R. China
