# Supplementary material for: Diversity and structural differences of bacterial microbial communities in rhizocompartments of desert leguminous plants
Source: PLoS One. 2020 Dec 22;15(12):e0241057. doi: 10.1371/journal.pone.0241057 (PMC7755220; doi:10.1371/journal.pone.0241057)
Supplement: Attachment — Submitted filename: Review_Comments.docx [file pone.0241057.s006.docx]

This work describes the microbial communities associated with three desert legume species, including two *Hedysarum* spp. and *Caragana microphylla*, as well as the various physicochemical properties of the soils form which they were isolated. An improvement of this work over similar studies referenced by the authors (*i.e.* Sun *et al.*) is the separation of under-shrub soils into “rhizocompartments” which include root tissue and associated soils in the rhizosphere. The methods used to isolate the samples, as well as the statistical analyses and soil property quantification approaches, are somewhat well-described but would be strengthened by adding some additional details as suggested below.

My main concern about this manuscript is that the interpretation of the statistical approaches, and of the perceived microbial community diversity, is inadequate. There are many cases in the manuscript (some of which I have highlighted below) whether the authors claim that “all” samples follow a certain trend or meet a certain significance level, when only the majority of samples do. Similarly, the authors’ methods for collecting DNA samples likely exclude any microbes that are not tightly associated to soil or roots, and the possible effects of this selection on the results are not discussed. These overstatements of the strength of the results make it difficult to have confidence in the authors’ interpretations.

**Specific comments**:

*Introduction*: The introduction is quite general, and provides little information about the specific plant species studied and what is already known about their associated microbiota. For example, it is mentioned that these species are legumes, yet it is not mentioned whether they engage in nitrogen-fixing symbioses with bacteria (which not all legumes do), and if so which bacterial symbionts are compatible, although there are many studies of this topic for *Hedysarum* spp. available in the literature. Some of this information is provided in the discussion, but giving more specific background for the chosen plant systems earlier in the paper will help readers contextualize the results.

*Methods*: It is unclear to me how the “inter-shrub bulk soil” was defined – how far away were the soil samples from the shrubs? The methods described for collection of the microbiota also do not seem to be optimized for maximum DNA sample recovery. Some questions that I had about the sample prep are:

- Was the soil DNA extraction kit also used for the root samples, or was a different extraction kit that is optimized for plant tissues used?
- How were the roots processed before extraction to break up the plant tissues (*e.g.* in a mortar and pestle or with enzyme treatment) – and were nodules included with the other root tissues?
- Why was the buffer that was used for ultrasonication of the roots, which likely includes many epiphytes that are more stably attached to roots as well as very fine soil particles, not added to the rhizosphere DNA sample?
- Did the authors test to see whether a 15 minute spin at 3200g is sufficient to collect the majority of microbes in the rhizosphere sample? In this method, anything that is not stably attached to the soil particles will be lost, since many *rhizobia*, for example, can’t be pelleted at such a low speed. I suspect the authors are losing quite a few microbes in their washes.

While it’s true that all extractions result in *some* sample loss, and many studies have followed similar protocols as the authors of this study, I recommend that the authors re-define their rhizocompartments based on the way the samples were processed to improve clarity. For example, the “rhizosphere soil” microbiota is perhaps better to define initially as “microbes strongly adhered to coarse root-associated soil” (although I think it is fine to use“rhizosphere soil” after this sample category is introduced).

*Results:* In virtually every main and supplemental, letters are used to represent groups of samples that are not statistically significant from one another, but specific p-values for comparisons between members of the “a”, “b”, “c”, *etc.* groups are never provided. The sample sizes are also not explicitly mentioned in the legends. This information should be added to the results, otherwise the strength of the results is very difficult to evaluate.

Additional comments:

- By “under-shrub” samples, do you mean the root, root zone and rhizosphere samples collectively? Please define.
- For Fig. 1, it would be helpful if each panel had different line widths or line types for each of the host species. Additionally, two different legends are provided for this figure (lines 251-260 and again on lines 261-265), which is confusing.
- In Fig. S1, a p-value is shown, but again I am not clear what this represents – which samples are being compared?
- Regarding the results presented in Table 1, in lines 271-273 of the manuscript the authors claim “Specifically, OTU number, Chao1 index, and Shannon index of rhizosphere soil samples for the two Hedysarum L. plants were all significantly higher than those for root samples and root zone soil samples, as well as those of inter-shrub bulk soil samples (P<0.05).” However, the rhizosphere soil samples are only significantly different from the inter-shrub bulk soils using the Chao1 index but not for the OTU number and Shannon index. Similarly, on lines 275-276, the authors claim “no significant effect of plant species on bacterial richness and diversity was observed” yet the root zone samples of species CM are indeed different from the root zone species of HM and HS using all three methods.
- In lines 217-319, the authors state “Specifically, Proteobacteria presented a significant step-by-step enrichment trend in the order of root>rhizosphere soil>root zone soil (P<0.01) under the three desert leguminous plant shrubs; Actinobacteria, Gemmatimonadetes presented a contrary trend (P<0.05)”. However, in Table S1, there is no difference in the Proteobacteria or Actinobacteria abundance between rhizosphere and root zone soils for sample HM, and there is no different between rhizosphere and root zone soils for Gemmatimonadetes for any of the plant species. There are similar overstatements of the results presented throughout the manuscript.
- For Fig. 5A-B, the raw data used to generate these diagrams should be provided in the supplement – or, if it is already provided in one of the tables, make it clear which one.
- For Fig. 6/Table S2, why pick the top ten rather than choose OTUs to define as “dominant” based on their relative abundance (*e.g.* anything comprising over X% of the total OTUs for all four samples)? I am also not sure what meaningful new information is added in Fig. 6 that is not already provided in Fig. 5C-D, other than including a specific representative species name. I suggest moving this tree to the supplement and shortening the corresponding section of the results.
- In Fig. 7, graphs are not labeled as (a) or (b).
