# Supplementary material for: Diversity and structural differences of bacterial microbial communities in rhizocompartments of desert leguminous plants
Source: PLoS One. 2020 Dec 22;15(12):e0241057. doi: 10.1371/journal.pone.0241057 (PMC7755220; doi:10.1371/journal.pone.0241057)
Supplement: Attachment — Submitted filename: Response to Reviewers.docx [file pone.0241057.s007.docx]

**Responses to all the reviewers’ comments on the manuscript:**

**“****Diversity and Structural Variability of Bacterial Microbial Communities in Rhizocompartments of Desert Leguminous Plants”**

**(****PONE-D-20-03936)**

**Submitted to *PLOS ONE* for publication**

**June 9, 2020**

**Response to Comments of Reviewer #1**

**Manuscript number**: PONE-D-20-03936

**Author(s)**: Ziyuan Zhou, Guodong Ding*, Minghan Yu*, Guanglei Gao,Yingying He

**Title**: Diversity and Structural Variability of Bacterial Microbial Communities in Rhizocompartments of Desert Leguminous Plants.

**General Comments:**

**This work describes the microbial communities associated with three desert legume species, including two *Hedysarum* spp. and *Caragana microphylla*, as well as the various physicochemical properties of the soils form which they were isolated. An improvement of this work over similar studies referenced by the authors (i.e. Sun et al.) is the separation of under-shrub soils into “rhizocompartments” which include root tissue and associated soils in the rhizosphere. The methods used to isolate the samples, as well as the statistical analyses and soil property quantification approaches, are somewhat well-described but would be strengthened by adding some additional details as suggested below.**

**My main concern about this manuscript is that the interpretation of the statistical approaches, and of the perceived microbial community diversity, is inadequate. There are many cases in the manuscript (some of which I have highlighted below) whether the authors claim that “all” samples follow a certain trend or meet a certain significance level, when only the majority of samples do. Similarly, the authors’ methods for collecting DNA samples likely exclude any microbes that are not tightly associated to soil or roots, and the possible effects of this selection on the results are not discussed. These overstatements of the strength of the results make it difficult to have confidence in the authors’ interpretations.**

**Response:**

We are very grateful to the reviewer for taking the time to read the manuscript, pointing out problems of crucial importance, and offering helpful comments and valuable opinions about how to improve the manuscript. Based on the reviewer’s opinions and suggestions, we have carefully and meticulously revised the manuscript. Specific revisions can be found in the revised manuscript submitted. It is our sincere hope that the revisions will address the shortages of the original manuscript. Provided below are detailed responses to the reviewer’s comments.

**Specific comments:**

**Introduction: The introduction is quite general, and provides little information about the specific plant species studied and what is already known about their associated microbiota. For example, it is mentioned that these species are legumes, yet it is not mentioned whether they engage in nitrogen-fixing symbioses with bacteria (which not all legumes do), and if so which bacterial symbionts are compatible, although there are many studies of this topic for Hedysarum spp. available in the literature. Some of this information is provided in the discussion, but giving more specific background for the chosen plant systems earlier in the paper will help readers contextualize the results.**

**Response:** This is consistent with the reviewer 4’s comments. Regarding this problem, we have conducted a literature search and incorporated relevant examples on nitrogen-fixing symbiosis of plants and rhizobia into the introduction section. Specifically, we have added the following sentences in the revised manuscript: ‘The role of rhizobia has always been the main research focus, and its most notable feature lies in its ability to establish a compatible interaction with molecules in the root of the host plant. This interaction depends on the expression of symbiosis-specific genes involving in gene compiling and signal output, and genes that can eventually trigger the unique organogenesis and other corresponding physiological responses of the host plants (Sablok et al., 2017). One study has shown that *Rhizobium sullae* is a highly efficient nitrogen-fixing rhizobium that can form symbiotic associations with *Hedysarum* plants (Yates et al., 2015). The bacteria cannot survive under extreme conditions to which *Hedysarum* plants can tolerate; however, it can survive in the host plants that are exposed to drought or alkaline conditions (Sablok et al., 2017). A study on genetic diversity of the rhizobium in plants of *Caragana* Fabr. in the Maowusu Sand land has revealed that when there is a shortage of water and nitrogen, the rhizobium may be considered as the basic functional unit of the local ecosystem (Gao et al., 2002). *Rhizobium alkalisoli* isolated from *Caragana* plants may promote the growth and environmental adaptation of the plants grown in saline-alkali arid areas (Li et al., 2009)’ (*Line: 70-82*).

**Methods: It is unclear to me how the “inter-shrub bulk soil” was defined – how far away were the soil samples from the shrubs? The methods described for collection of the microbiota also do not seem to be optimized for maximum DNA sample recovery.**

- Response: ‘Inter-shrub bulk soil’ collected in this study is the soil between two adjacent shrubs (about 2 m apart), where plants do not grow, and its sampling depth was the same as that of the root zone soil. (*Line: 144-146*)
- Response: Regarding this concern, we have performed the collection based on the procedures described in Xiao et al. (2017). The reviewer is correct that the DNA sample loss caused by the operational errors is inevitable; we thus have also taken these errors into account. In our future research, we will optimize the sampling and experimental processes by using more advanced equipment and technology, and will try to minimize unnecessary errors and losses to the greatest extent possible.

**Some questions that I had about the sample prep are:**

**Was the soil DNA extraction kit also used for the root samples, or was a different extraction kit that is optimized for plant tissues used?**

- Response: We did not run the additional process that is specific for samples of rhizocompartments, and specially optimized extraction kits for specific plant tissues were also not used. Instead, we used E.Z.N.A. soil DNA kits (OMEGA, United States) for both types of samples.

**How were the roots processed before extraction to break up the plant tissues (e.g. in a mortar and pestle or with enzyme treatment) – and were nodules included with the other root tissues?**

- Response: In fact, after collecting and cleaning, the grouped samples were sent to a third party, the Beijing Allwegene Technology Co., Ltd. (http://www.allwegene.com), which is a biotechnology company located in China, for subsequent extraction and evaluation. The detail of evaluation and processing information, such as the methods and materials, was provided by the company as well. To response to the questions raised by the reviewer, we have contacted the company and their responses were as follows: ‘The DNA extraction processes of plant tissue and soil samples were carried out in strict accordance with the manufacturer’s instructions. Before DNA extraction, the plant root samples were frozen in liquid nitrogen and crushed in a mortar. The extraction of DNA from all samples (0.5 g each) presented in this study was carried out using E.Z.N.A. soil DNA kits (OMEGA, United States). The extracted genomic DNA was stored at -80 °C until subsequent use.’ (*Line: 196-200*)
- Response: Root samples were collected from secondary or tertiary root branches without nodules. Healthy and intact root samples (5-8 cm in length) with similar diameters were cut and stored in sterile sample bags to avoid contaminations from nodule endophytes and other foreign bacteria. (*Line: 147-150*)

**Why was the buffer that was used for ultrasonication of the roots, which likely includes many epiphytes that are more stably attached to roots as well as very fine soil particles, not added to the rhizosphere DNA sample?**

**Did the authors test to see whether a 15 minute spin at 3200g is sufficient to collect the majority of microbes in the rhizosphere sample? In this method, anything that is not stably attached to the soil particles will be lost, since many rhizobia, for example, can’t be pelleted at such a low speed. I suspect the authors are losing quite a few microbes in their washes.**

**While it’s true that all extractions result in some sample loss, and many studies have followed similar protocols as the authors of this study, I recommend that the authors re-define their rhizocompartments based on the way the samples were processed to improve clarity. For example, the “rhizosphere soil” microbiota is perhaps better to define initially as “microbes strongly adhered to coarse root-associated soil” (although I think it is fine to use“rhizosphere soil” after this sample category is introduced).**

- Response: This was performed according to the methods described in Xiao et al. (2017) without any additional steps and operations. We completely agree with the reviewer that this procedure may cause analysis errors and sample loss, and it is indeed a shortcoming of our study. Thus, in our future experiment, we will pay more attention to the optimization of our procedure. As per the reviewer’s suggestion, we have also revised the definition of ‘rhizosphere soil microbiota’ to ‘microbes that are strongly adhered to the root-associated soil’ and have added it to the revised manuscript (*Line: 157-158*).

**Results: In virtually every main and supplemental, letters are used to represent groups of samples that are not statistically significant from one another, but specific p-values for comparisons between members of the “a”, “b”, “c”, etc. groups are never provided. The sample sizes are also not explicitly mentioned in the legends. This information should be added to the results, otherwise the strength of the results is very difficult to evaluate.**

- Response: We totally agree with the reviewers’ comments, and please see our point-to-point responses below.

**Additional comments:**

**By “under-shrub” samples, do you mean the root, root zone and rhizosphere samples collectively? Please define.**

- Response: We thank the reviewer for this suggestion, which makes the description to be more easily understood. The following was added to the revised manuscript: ‘In this study, we selected three common local dominant xeric leguminous shrub species,, namely *Caragana microphylla*, *Hedysarum mongolicum*, and *Hedysarum scoparium*, and studied the changes of bacterial community diversity in four rhizocompartments of their microbiota, including three types of under-shrub samples (i.e., root, rhizosphere soil, root zone soil) and inter-shrub bulk soil.’ (*Line: 125-129*)

**For Fig. 1, it would be helpful if each panel had different line widths or line types for each of the host species. Additionally, two different legends are provided for this figure (lines 251-260 and again on lines 261-265), which is confusing.**

- Response: As suggested by reviewers 1 and 4, we have redrawn Figure 1 and corrected the relevant errors in the revised manuscript. (*Line: 284-292*)

**In Fig. S1, a p-value is shown, but again I am not clear what this represents – which samples are being compared?**

- Response: We have re-analyzed the data and prepared Table 2 and Fig. S1, which are associated with ANOSIM. We completely understand the concern of the reviewer 4’s concern regarding the Bray-Curtis Distance-based OTU clustering; thus, we have followed the reviewers' advice and performed the UNIFRAC-based ANOSIM analysis. The results of this analysis can be found in the ANOSIM Table 2 and Fig. S1 in the revised manuscript.

In order to avoid the interference between Figure A and Figure B in Fig. S1, this new ANOSIM figure focuses only on the comparison of the four proposed type of rhizocompartments, and the original settings of image A was removed.

**Regarding the results presented in Table 1, in lines 271-273 of the manuscript the authors claim “Specifically, OTU number, Chao1 index, and Shannon index of rhizosphere soil samples for the two Hedysarum L. plants were all significantly higher than those for root samples and root zone soil samples, as well as those of inter-shrub bulk soil samples (P<0.05).” However, the rhizosphere soil samples are only significantly different from the inter-shrub bulk soils using the Chao1 index but not for the OTU number and Shannon index. Similarly, on lines 275-276, the authors claim “no significant effect of plant species on bacterial richness and diversity was observed” yet the root zone samples of species CM are indeed different from the root zone species of HM and HS using all three methods.**

- Response: Regarding the analysis error in Table 1, which is similar to the comment from reviewer 4, we have changed the content in lines 271-273 to: ‘for the two *Hdysarum* species, the number of OTU, as well as their Chao1 and Shannon values of the samples collected in the rhizosphere soil were significantly higher than those of samples collected in the root and soil samples in the root zone.’ (modified in *line: 310-312*)
- Response: Regarding the erratic discussion in lines 275-276, we modified it to: Plant species and plant rhizocompartments jointly affect the alpha diversity of soil bacterial communities under and between shrubs, but rhizocompartments play a leading role (Table 1). (modified in *line: 314-316*)

**In lines 317-319, the authors state “****Specifically, Proteobacteria presented a significant step-by-step enrichment trend in the order of root>rhizosphere soil>root zone soil (P<0.01) under the three desert leguminous plant shrubs; Actinobacteria, Gemmatimonadetes presented a contrary trend (P<0.05)”. However, in** **Table S1, there is no difference in the Proteobacteria or Actinobacteria abundance between rhizosphere and root zone soils for sample HM, and there is no different between rhizosphere and root zone soils for Gemmatimonadetes for any of the plant species. There are similar overstatements of the results presented throughout the manuscript.**

- Response: We thank the reviewer for pointing this out. The inappropriate description of Table S1 here has been corrected in the revised manuscript. We have added the following sentence to the manuscript: ‘Specifically, for Proteobacteria, the enrichment of *C. microphylla* and *H.scoparium* shrubs had the same trend: root > rhizosphere soil > root zone soil (*P* < 0.01), whereas the enrichment of Actinobacteria had the opposite trend (*P* < 0.05).’ (*Line: 356-358*)

**For Fig. 5A-B, the raw data used to generate these diagrams should be provided in the supplement – or, if it is already provided in one of the tables, make it clear which one.**

- Response: For this point, we have considered the comments from both the reviewers 1 and 4, re-analyzed the data, and prepared a new Figure 5. We have also organized the related statistical data into a single Excel file and submitted it as a new Table S2. This has also been explained in the legend of Figure 5 (*Line: 411-412*). The description of Table S2 and Figure 5 here has been corrected in *lines 416-426*.

**For Fig. 6/Table S2, why pick the top ten rather than choose OTUs to define as “dominant” based on their relative abundance (e.g. anything comprising over X% of the total OTUs for all four samples)? I am also not sure what meaningful new information is added in Fig. 6 that is not already provided in Fig. 5C-D, other than including a specific representative species name. I suggest moving this tree to the supplement and shortening the corresponding section of the results.**

- Response: We thank the reviewer for this suggestion. We have prepared figure and table, and performed a complete analysis following the methods described in Beckers et al. (2017) entitled ‘Structural variability and niche differentiation in the rhizosphere and endosphere bacterial microbiome of field-grown poplar trees’. In this paper, the authors defined the ‘core bacterial microbiome’ as the most abundant 10 OTUs in each biological niche. In another paper by Wang et al. (2019), the authors also employed the same method to study the core bacterial microbiomes of different biological niches of transgenic poplar plants, and performed indicator species analysis (*Line: 260-261*). In our study, we adapted the same method to explore the core bacterial microbiomes in the root microdomains of legumes, and our data should be beneficial for preparing specific bacterial agents that can promote plant growth and improve the quality of soil used to grow these legume plants. This can be of significance for the ecological restoration of deserts, which in fact is the ultimate purpose of our study. The new idea proposed by the reviewer is undoubtedly feasible, and we will be sure to adopt it in our future research. In our opinions, we believe that the current method and analysis do not cause serious issues; thus, we would like to keep them in the present manuscript. Nevertheless, we have followed the second part of the suggestion, by which we have reduced the analysis of this part and moved the results to supplementary materials.

The genus of bacteria in the legumes shown in Figures 5C-D were enriched and filtered. The decreasing/enriching trends of bacteria in the legume plants (Figs. 5C and 5D) were listed based on their relative abundance in each rhizocompartments, which indicate the accuracy of the analysis results are presented in Figures 5A and 5B. The related dataset has also been integrated into a new raw data file (new Table S2) for Figure 5. Once again, as we strongly believe that this part is of high importance, we would like to kindly ask for the reviewer’s understanding.

**In Fig. 7, graphs are not labeled as (a) or (b).**

- Response: The error raised by the reviewer has now been corrected (please see Fig. 6).

**Thanks again for the reviewer’s help to improve the manuscript.**

**Reference:**

Beckers B, Op De Beeck M, Weyens N, Boerjan W, Vangronsveld J. Structural variability and niche differentiation in the rhizosphere and endosphere bacterial microbiome of field-grown poplar trees. Microbiome. 2017; 5: 25. <https://doi.org/10.1186/s40168-017-0241-2>

Gao, L. F., Hu, Z. A., & Wang, H. X. (2002). Genetic diversity of rhizobia isolated from *Caragana intermedia* in Maowusu sandland, north of China. Letters in Applied Microbiology, 35(4), 347–352. <https://doi.org/10.1046/j.1472-765x.2002.01192.x>

Beckers B, Op De Beeck M, Weyens N, Boerjan W, Vangronsveld J. Structural variability and niche differentiation in the rhizosphere and endosphere bacterial microbiome of field-grown poplar trees. Microbiome. 2017; 5: 25. <https://doi.org/10.1186/s40168-017-0241-2>

Li Lu, Y., Chen, W. F., Li Han, L., Wang, E. T., & Chen, W. X. (2009). *Rhizobium alkalisoli* sp. nov., isolated from *Caragana intermedia* growing in saline-alkaline soils in the north of China. Int J Syst Evol Microbiol. 59 (12): 3006-11. <https://doi.org/10.1099/ijs.0.007237-0>

Sablok G, Rosselli R, Seeman T, van Velzen R, Polone E, Giacomini A, La Porta N, Geurts R, Muresu R and Squartini A. (2017) Draft Genome Sequence of the Nitrogen-Fixing *Rhizobium sullae* Type Strain IS123^T^ Focusing on the Key Genes for Symbiosis with its Host *Hedysarum coronarium* L. Front. Microbiol. 8:1348. <https://dx.doi.org/10.3389%2Ffmicb.2017.01348>

Xiao X, Chen W, Zong L, Yang J, Jiao S, Lin Y, Wang E, Wei G. Two cultivated legume plants reveal the enrichment process of the microbiome in the rhizocompartments. Mol Ecol. 2017; 26(6): 1641–1651. <https://doi.org/10.1111/mec.14027>

Yates, R., Howieson, J., De Meyer, S. E., Tian, R., Seshadri, R., Pati, A., Reeve, W. (2015). High-quality permanent draft genome sequence of *Rhizobium sullae* strain WSM1592; a *Hedysarum coronarium* microsymbiont from Sassari, Italy. Standards in Genomic Sciences, 10(1). <https://doi.org/10.1186/s40793-015-0020-2>

**Response to Comments of Reviewer #2**

**Manuscript number**: PONE-D-20-03936

**Author(s)**: Ziyuan Zhou, Guodong Ding*, Minghan Yu*, Guanglei Gao,Yingying He

**Title**: Diversity and Structural Variability of Bacterial Microbial Communities in Rhizocompartments of Desert Leguminous Plants.

**General Comments:**

**The manuscript titled as "Diversity and Structural Variability of Bacterial Microbial Communities in Rhizocompartments of Desert Leguminous Plants" compared microbiome from four compartments, and found root microbiomes had higher structural variabilities than the other 3 types of microbiomes.**

**Response:**

Thanks to the reviewer for sparing time to go through the manuscript, highlighting very important issues and providing helpful comments and valuable suggestions to improve the manuscript. We have revised the manuscript seriously and carefully according to the reviewer’s comments and suggestions. Specific changes could be found in the revised manuscript now submitted. We would like that it could offset the shortcomings in the original manuscript. More details and point-to-point responses to the reviewer’s comments are listed as follows.

**Major concern: The comparison of four compartments is interesting. However, ANOSIM table is missing, therefore it is hard for the reviewer to determine whether the conclusion is solid for the structural variability. Please present the table which should contain the R and P-value. To avoid confusion, a summary akin to table 2 in this other paper** <https://link.springer.com/article/10.1186/s40168-017-0241-2> **is the one missing.**

- Response: As mentioned by the reviewer, adding quantitative ANOSIM analysis is critical for our study, and a lack of this analysis is the major shortcoming of our work. As suggested by the reviewers 1, 2 and 4, we have added the ANOSIM analysis based on our UNIFRAC distance calculation. We have prepared a table tabulating the *r* and *P* values obtained from ANOSIM analysis (Table 2 in the revised manuscript) and a new Fig. S1. These analyses were used to quantify the characteristics of the hierarchical changes of bacterial microbial community structure in the four root microdomains of the xerophytic legumes under and inter shrubs. Regarding the other weakness raised by the reviewers, we have thoroughly revised and improved the manuscript accordingly.

| Table 2 Analysis of similarity (ANOSIM) | | | | | |  |
| --- | --- | --- | --- | --- | --- | --- |
| Phylogenetic level |  | Phylum | |  | OTU | |
| ANOSIM output |  | *r* | *P-value* |  | *r* | *P-value* |
| R vs. RS |  | 0.9499 | 0.001 |  | 0.9057 | 0.001 |
| R vs. RZ |  | 0.9952 | 0.001 |  | 0.9585 | 0.001 |
| RS vs. RZ |  | 0.4366 | 0.004 |  | 0.2541 | 0.023 |
| R vs. Bulk |  | 1 | 0.001 |  | 0.987 | 0.001 |
| RS vs. Bulk |  | 0.749 | 0.001 |  | 0.4784 | 0.001 |
| RZ vs. Bulk |  | 0.0706 | 0.137 |  | 0.1022 | 0.08 |
| Rhizocompartments |  | 0.6426 | 0.001 |  | 0.4953 | 0.001 |
| Plant rhizocompartments effects on the microbial community structures were calculated using analysis of similarities (ANOSIM) based on the Weighted UniFrac distance metric (999 permutations).  R: root; RS: rhizosphere soil; RZ: root zone soil; Bulk: inter-shrub bulk soil.  Significance levels: *P* ≤ 0.05; *P* ≤ 0.01. | | | | | |  |
|  |  |  |  |  |  |  |
|  |  |  |  |  |  |  |

**Minor comments:**

**Even though the written English is intelligible, a thorough proof-reading is highly recommended.**

- Response: We thank the reviewer for this suggestion. To improve the English language of the manuscript, we have submitted our manuscript to a language editing agency for proofreading and editing.

**The resolution of the figures are poor, and the reviewer could not download tables as it shows to be corrupted. Therefore, it was hard to determine the clustering in Figure 2B. Figure 4 letters for significance are hard to distinguish.**

- Response: We sincerely apologize for the inconvenience that was caused by the image quality problem. We have enhanced the image resolution of Figure 2 and 4; it now has a higher image resolution. We hope that the newly submitted figures meet the requirements of the journal and satisfy the reviewers.

**Response to Comments of Reviewer #3**

**Manuscript number**: PONE-D-20-03936

**Author(s)**: Ziyuan Zhou, Guodong Ding*, Minghan Yu*, Guanglei Gao,Yingying He

**Title**: Diversity and Structural Variability of Bacterial Microbial Communities in Rhizocompartments of Desert Leguminous Plants.

**General Comments:**

**In general, the topic of the Diversity and Structural Variability of Bacterial Microbial Communities in Rhizocompartments of Desert Leguminous Plants is an important area to develop better understanding. Much of the actual work performed on the manuscript and general analysis was adequate. However, as pointed below, the manuscript contains some problems, improper data interpretation, data presentation, and discussion. These need to be revised carefully.**

**Response:**

Thanks for the helpful comments and suggestions. We have revised the manuscript seriously and carefully according to the reviewer’s comments and suggestions, and carefully checked the whole manuscript and revised some typos. We hope it could offset the shortcomings in the original manuscript. More details and point-to-point responses to the reviewer’s comments are listed as follows.

**Comments for abstract:**

**I am not quite sure the format of abstract published by Plos One, however, the results and conclusion in the abstract need to include meaningful data. For example, line 26 “had significant effects on …”, what does this “significant effects” mean? It could be positive or negative effect, and “specific bacterial microbiomes”, what is this “specific bacterial microbiomes” mean? And line 35-36, “while soil physicochemical factors have a significant influence”, what is this significant influence? I would recommend the author rewrite this abstract.**

- Response: We appreciate the reviewer for pointing this out. We have referred to the abstract writing guideline of the *PLOS ONE* and have rewritten the abstract according to the reviewer’s suggestion. (*Line: 9-28*)

**Line 63: there is an error, the meaning of this sentence is not clear, “improve soil” or “improve soil quality/health”?**

- Response: The meaning of the original sentence here should be interpreted as: ‘the well-distributed root system of the plant can fix the soil under the shrub and improve the soil texture; that is, it ‘improves the soil quality’. (*Line: 54*)

**Line 68: “participate in and control many processes of soil ecosystems” need to rewrite, here might be some errors. Probably “participate in and control many soil ecosystem functioning processes” is better to understand.**

- Response: We thank the reviewer very much for these careful and detailed suggestions. The proper word choice here is highly important for the more accurate explanation of the relevant research cited in this article. Therefore, we agree with the reviewer's suggestion and have changed the text to ‘participate in and control many soil ecosystem functioning processes’. (*Line: 63-64*)

**Line 71: citation needed here.**

- Response: Citations have been correctly added in the revised manuscript. (*Line: 68*)

Devine TE, Kuykendall LD. Host genetic control of symbiosis in soybean (Glycine max L). Developments in Plant and Soil Sciences. 1996; 186: 173–187. <https://doi.org/10.1007/978-94-011-5700-1_24>

**Line 81: “30,000 prokaryotes per gram of root” does this mean 30,000 species or this is the number? If the author means how many species in each gram of root soil, please indicate it here.**

- Response: We thank the reviewer for pointing this out. We agree that the citation in the original text was not sufficiently accurate; therefore, we have re-read the citations and re-written this part. (*Line: 89*)

**Line 148: “corresponding” might be better than “according”.**

- Response: We agree with the reviewer's comments and have revised the manuscript accordingly. (*Line: 168*)

**Line 251-255: Don’t need figure cations here. This is a duplicate as below line 261-265.**

- Response: We thank the reviewer for these comments. We have made the changes in the revised manuscript correspondingly.

**Table 1: Error in the first row, third column, the title for OTU.**

- Response: We thank the reviewer for these comments. We have revised the Table 1 accordingly.

**Line 272: use “both” instead of “all”.**

- Response: We agree with the reviewer's comments and have revised the manuscript accordingly. (*Line: 310-312*)

**Line 278-285: there is no need to explain what analysis you did for your sample in the results part, you may explain in materials and methods. I would suggest the author consider remove it. Just state what significant results you get from your sequencing data.**

- Response: As suggested by the reviewers 3 and 4, we have moved the reduced version of the description of the analysis method to the Materials and Methods section. Regarding the erroneous description that was pointed out by the reviewer, we have deleted this description.

**Line 303: use “r” instead of “R”.**

- Response: We agree with the reviewer's comments and have revised the manuscript accordingly. (*Line: 343*)

**Line 305: there is a mistake in this sentence.**

- Response: As advised, we have deleted ‘on’ in this sentence. (*Line: 344*)

**Line 311: figure 4A and 4B are duplicate in figure 3, why does the author create these two graphs? If the author want to show results at phylum, order, and genus levels, please create another graph at genus level use sequencing data as showed in figure 3.**

- Response: We thank the reviewer for pointing out this issue and for suggestions for better modification of the manuscript. Figure 4 shows the relative abundance difference of major contributing bacterial groups at the Phyla, Order, and Genus levels, and the figure displays the significant enrichment of each bacterial group at various taxonomic levels in the four rhizocompartments. Yet, Figure 3 only shows the species composition of the major contributing groups at the Phyla and Order levels, but does not show the significant differences in relative abundance of the dominant bacterial groups among all the rhizocompartments. We have great respect for all the reviewer’s suggestions; however, to ensure that the interpretation of the data is accurate, we have taken into account the comment regarding Figure 4 from reviewer 2 and only revised the significant marks of the figure. We do hope that the reviewer understand our intent.

**Line 313: format error at the end of the line.**

- Response: We thank the reviewer for these comments. We have made the changes in the revised manuscript correspondingly. (*Line: 352*)

**Line 344-345: Error in this sentence, please rewrite.**

- Response: We have changed the sentence to: ‘Although our results also indicated that the enrichment trend of *Rhizobium* in *C. microphylla* roots (*P* < 0.05) and that of *Massilia* in *H. mongolicum* roots (*P* < 0.01) were most significant, some similar trends were also manifested in other major contributing bacterial genera in the four rhizocompartments’. (*Line: 381-384*)

**Before discussion in Table 2, first column, use “****P-value” instead of “P”.**

- Response: We thank the reviewer for these comments. We have made the changes in the revised manuscript correspondingly. (please see Table 3)

**Line 482: need to indicate which figure represent this result, so people are easier to read and understand the manuscript.**

- Response: To address this point, we have followed the reviewer’s comments and added the legends and table notes of the corresponding supporting data at the end of the sentence. (*Line: 577*)

**Line 481-489: I understand the author would like to explain and discuss why bacterial communities in roots have a higher structural variability compared to soil bacterial communities. However, the author only provided several research findings from Gottel et al, Bulgarelli et al, Lundberg et al. and Peiffer et al, I don’t see any evidence that can support the author’s point here. Instead, if the author could provide support on line 490-491, then it will be easier to understand.**

- Response: We completely agree with the reviewer’s suggestion. To overcome the shortcoming of this corroboration, we have provided more relevant citations and added the following sentences to the revised manuscript: ‘In fact, for a), the bacteria associated with plants must be adapted to environmental variations (such as humidity, pH, and nutrient acquisition) in the micro-niche of rhizocompartments (Narteh et al. 1997; Xiao 2017B), and only the highly competitive bacteria can successfully colonize the root system (Beckers et al. 2017; wang et al. 2019); and for b), the intricate interaction between soil-borne bacteria and the host plant’s immune system (Jones et al. 2006; Hardoim et al. 2008; Compant et al. 2010; Bulgarelli et al. 2012; Turner et al. 2013) are dependent on the colonization ability and characteristics of endophytes (Hardoim et al. 2008; Reinhold-Hurek et al. 2006); thus, the migration and colonization of certain soil bacteria in the root system are highly variable processes.’ (*Line: 508-514*)

**Line 574: Error in this sentence “formed had low richness and diversity”.**

- Response: We thank the reviewer for pointing this out. We have changed the sentence to: ‘Although the root endophyte microbiomes had low richness and diversity, their structural variability was highest among all the four rhizocompartments.’ (*Line: 602-604*)

**In supporting materials: S4 table title “S4 Table. Soil physicochemical properties of rhizospheres and root zones under the two shrub species (n = 3).” I think there are three shrub species. And the unit for TP is not correct.**

- Response: We very much appreciate the reviewer for pointing out this mistake, which has now been revised. In addition, we have verified and changed the unit of total phosphorus (TP) from k\kg into g\kg. (please see S5 Table)

**Thanks again for the reviewer’s help to improve the manuscript.**

**Reference:**

Beckers B, Op De Beeck M, Weyens N, Boerjan W, Vangronsveld J. Structural variability and niche differentiation in the rhizosphere and endosphere bacterial microbiome of field-grown poplar trees. Microbiome. 2017; 5: 25. <https://doi.org/10.1186/s40168-017-0241-2>

Bulgarelli D, Rott M, Schlaeppi K, Ver Loren van Themaat E, Ahmadinejad N,Assenza F, et al. Revealing structure and assembly cues for Arabidopsis root-inhabiting bacterial microbiota. Nature. 2012;488:91–5. <https://doi.org/10.1038/nature11336>

Compant S, Clément C, Sessitsch A. Plant growth-promoting bacteria in the rhizo- and endosphere of plants: Their role, colonization, mechanisms involved and prospects for utilization. Soil Biol Biochem. 2010; 42: 669–78. <https://doi.org/10.1016/j.soilbio.2009.11.024>

Hardoim PR, van Overbeek LS, Van Elsas JD. Properties of bacterial endophytes and their proposed role in plant growth. Trends Microbiol. 2008; 16: 463–71. <https://doi.org/10.1016/j.tim.2008.07.008>

Jones J, Dangl J. The plant immune system. Nature. 2006; 444: 323–329. <https://doi.org/10.1038/nature05286>

Narteh LT, Sahrawat KL. Potentially mineralizable nitrogen in West African lowland rice soils. Geoderma. 1997; 76(2): 145-154. <https://doi.org/10.1016/S0016-7061(96)00097-3>

Reinhold-Hurek B, Maes T, Gemmer S, Van Montagu M, Hurek T. An endoglucanase is involved in infection of rice roots by the not-cellulose-metabolizing endophyte Azoarcus Sp. strain BH72. Mol. Plant Microbe Interact. 2006; 19: 181–188. <https://doi.org/10.1094/MPMI-19-0181>

Turner, T.R., James, E.K. & Poole, P.S. The plant microbiome. Genome Biol 14, 209 (2013). <https://doi.org/10.1186/gb-2013-14-6-209>

Xiao X, Fan MC, Wang ET, Chen WM, Wei GH. Interactions of plant growth-promoting rhizobacteria and soil factors in two leguminous plants. Appl Microbiol Biotechnol. 2017; 101: 8485–97. https://doi.org/10.1007/s00253-017-8550-8

Wang, Y.B., Zhang, W.X., Ding, C.J., Zhang, B.Y., Huang, Q.J., Huang, R.F., Su, X.H. (2019). Endophytic Communities of Transgenic Poplar Were Determined by the Environment and Niche Rather Than by Transgenic Events. Front. Microbiol, 26. <https://doi.org/10.3389/fmicb.2019.00588>

**Response to Comments of Reviewer #4**

**Manuscript number**: PONE-D-20-03936

**Author(s)**: Ziyuan Zhou, Guodong Ding*, Minghan Yu*, Guanglei Gao,Yingying He

**Title**: Diversity and Structural Variability of Bacterial Microbial Communities in Rhizocompartments of Desert Leguminous Plants.

**General Comments:**

**In the manuscript “Diversity and Structural Variability of Bacterial Microbial Communities in Rhizocompartments of Desert Leguminous Plants” the authors aimed to characterize the microbial communities found in, on, near, and further from the roots of several shrub species in a particular desert habitat to determine if shrubs influence the microbial communities. The authors calculate metrics of alpha diversity and find that root endophytic samples were least diverse, but that root zone soil is more diverse than between-shrub soil. – i.e. shrubs influence microbial communities. The sampling and sequencing methods are appropriate for this goal, although I have noted a few questions that came up in line comments. I commend the authors on these aspects.**

**However, I have a few major concerns about the analyses, and one about the writing and organization. Briefly, while some analyses were good, I found others redundant, some poor quality, and a key analysis to support a conclusion seemingly missing. I found the text on data analysis in the methods greatly lacking in detail, with some misplaced to the results, and not all of the discussion and conclusions are well-linked to the data. Note that lack of clarity could partly explain some of my concerns about the analyses. While my concerns may seem grievous, because the manner of data collection and sequencing appears fundamentally valid, I believe the authors could address all my concerns.

Major Comments:**

**I believe the authors wished to quantify how variable communities from certain sample types were WITHIN that type. This is what I think the authors mean by “structural variations” and similar language throughout the manuscript. Conclusions about this are reported (“we found that the formation of bacterial microbial communities in root was a higher variability process”), but I see no clear test of this. For example, species turnover (beta diversity) between samples within a type appears to not be calculated, or it isn't explained. There are various appropriate calculations of beta diversity the authors could calculate with the data they have. Instead, two redundant metrics identifying compositional change ACROSS sample types are employed. Both PCA and hierarchical clustering of Bray-curtis distance are used, and both show that endophytic communities differ from the other sample types, but that the remaining sample types and all species are similar in species composition to each other (though I wonder if there are subtle differences, as I note below). These are useful analyses, but not sufficient to support the conclusion quoted above.**

**Likewise, the authors use several methods to ask which species differ across sample types (e.g. which species drive the result above). First, they could have discussed which taxa contribute to the axes in the PCA they already ran, but instead chose to use totally different analyses. They cut the dataset to more abundant groups (not sure exactly how, methods are sparse, but this is repeated across cases when grouping by phyla, order, or genus), then individually tested changes in relative abundance of each group across sample types. This ignores a major constraint – 16s rDNA is relative abundance, so changes in one group are NOT independent of others (when one increases in relative abundance, others must therefore decrease, even if absolute abundance in these others in the field has not changed). Therefore, taxa-by-taxa tests for significant trends do not make sense. Instead, I suspect the third analysis (if it had been more fully explained) might do just this: it appears to me from the text that the metastats packages looks for groups of OTUs with correlated changes in abundance, i.e. acknowledging that each change OTU abundance is not independent from the others. I would recommend the authors develop the results of this test more (which taxa are in correlated groups?), or use the correlated changes in OTUs from the PCA, and skip the other tests. Lastly, the authors define conditions for a core microbiome, and again test where it is enriched – with the circular result that the most abundant taxa from a set of samples are enriched in that sample. I may be missing something in the methods (see below), but if not, this circular analysis is not very helpful and should be removed from the manuscript, along with figure 6.**

**The authors investigate how soil physiocochemical factors differ across sample types. This analysis largely made sense. The authors also checked to see if some bacterial groups were correlated with these factors – my suggestion here is to use the full dataset rather than the core microbiome only – the core are the set of microbes that are stable across samples, and so the LEAST likely to be influenced by soil properties.**

**My last major comment is about the organization of writing and clarity of logic. Much data analysis text is both insufficient and found in the results instead of the methods. The data analysis section is not co-linear with how the tests were run or how the results were presented. The discussion section also varies in quality. Some places appropriately reference both the results and the literature to support conclusions (the first two paragraphs after the heading “Drivers of the differentiation of bacterial communities under desert leguminous plant shrubs” are great, for example). But the rest of the discussion is missing those key aspects. Given the places where the writing is is clear and does make sense (see referenced paragraphs in discussion, and the rest of the methods is much easier to follow), I expect the authors can fix this.**

**The data dryad link doesn't work for me, though I believe the authors that they have deposited their data, and expect there is a simple typo. Analysis scripts should be made available along with the data.**

**I have included line-referenced comments below, since I took the time to note them down when reading. Given the sweeping nature of the changes I recommended above, I do not know how useful these specifics will be to the authors or editors.**

**Response:**

Thank you very much for giving us an opportunity to revise our manuscript. We appreciate the editor and reviewers very much for their constructive comments and suggestions on our manuscript entitled "Diversity and Structural Variability of Bacterial Microbial Communities in Rhizocompartments of Desert Leguminous Plants". We have studied reviewers’ comments carefully. According to the reviewers’ detailed suggestions, we have made a careful revision on the original manuscript. A marked-up copy of our manuscript that highlights changes made to the original version, which we would like to submit for your kind consideration.

**Introduction**

**Line 56: It is a shortcut to say “The nitrogen-fixing ability of leguminous plants”, as it is associated microbes that fix nitrogen. The authors are clearly aware of this, so I assume this is a typo.**

- Response: We greatly appreciate the reviewer for the comments. In the revised manuscript, we have added the following sentence: “The symbiosis between legumes and rhizobia is particularly important for biological nitrogen fixation. It not only provides important biological nitrogen source to agriculture and natural ecosystems, but also is conducive to the sustainable production of agriculture by providing necessary ecological services that are of great significance for increasing soil fertility (Tang et al. 2008).” (*Line: 46-49*)

**Lines 53-60: This paragraph seems less important towards motivating the study than the other points. The authors might cut this and enhance other points.，．．．**

- Response: We agree with the reviewer’s suggestion. We have therefore removed some meaningless descriptions and added new points that are more valuable and motivating to show the significance of this study. The following was added: “Legumes have high species diversity and adaptability in various global ecosystems (LPWG, 2013; Liu et al. 2017); these superiorities are largely due to the unique symbiotic interaction between rhizobia and legumes (Young et al., 2006; Giraud et al., 2007; Wang et al., 2012). (*Line: 44-45*)

**Line 66: Here and elsewhere, I think the authors may be accidentally mis-using “adaptability”, or they have not explained fully. Adaptability of desert vegetation would be if desert plants had a greater capacity to evolve (genetically) in response to selection pressures than other plants. There could be a literature stream on this of which I am not aware. However, I think the authors actually mean to communicate that desert vegetation has a high tolerance of extreme abiotic conditions.**

- Response: We thank the reviewer for this suggestion. To better explain this point, we have added the following detail, which was obtained from the relevant literature, into the revised manuscript: ‘Rhizobia inoculation and organic improvers are widely used in the vegetation restoration and soil quality improvement of the degraded Mediterranean area (Alguacil et al., 2003; Azcon et al., 2009; Caravaca et al., 2002; Medina et al., 2004). The addition of bacteria can increase the proliferation and development of natural microbes in soil in arid regions, and can subsequently promote soil enzyme activities (Caravaca et al., 2005) and increase a supply of low-mobility nutrients, such as potassium and phosphorus, in the soil, which can in turn promote plant growth (Caravaca et al., 2003; Bashan et al., 2004).’ (*Line: 58-63*)

**Line 71:** **“believed” maybe isn't quite the right word. No one believes they are completely co-transmitted like a true genome, and I don't think that's what the authors mean here?**

- Response: We thank the reviewer for pointing this out. Accordingly, we have changed ‘believed’ to ‘considered’. (*Line: 67*)

**Line 72: This is an example of where specifics would help the writing. It's not totally clear what's meant by “The microbiome not only provides functional assistance and support to host plants” unless the reader has background knowledge already.**

- Response: This is consistent with the reviewer 1’s comments. With regard to this shortcoming, we have incorporated some background on the establishment of nitrogen-fixing symbiota in the relevant plant species and rhizobia, as well as some examples of mutual symbiosis. The following sentences were added: The role of rhizobia has always been the main research focus, and its most notable feature lies in its ability to establish a compatible interaction with molecules in the root of the host plant. This interaction depends on the expression of symbiosis-specific genes involving in gene compiling and signal output, and genes that can eventually trigger the unique organogenesis and other corresponding physiological responses of the host plants (Sablok et al., 2017). One study has shown that *Rhizobium sullae* is a highly efficient nitrogen-fixing rhizobium that can form symbiotic associations with *Hedysarum* plants (Yates et al., 2015). The bacteria cannot survive under extreme conditions to which *Hedysarum* plants can tolerate; however, it can survive in the host plants that are exposed to drought or alkaline conditions (Sablok et al., 2017). A study on genetic diversity of the rhizobium in plants of *Caragana* Fabr. in the Mu Us Desert has revealed that when there is a shortage of water and nitrogen, the rhizobium may be considered as the basic functional unit of the local ecosystem (Gao et al., 2002). *Rhizobium alkalisoli* isolated from *Caragana* plants may promote the growth and environmental adaptation of the plants grown in saline-alkali arid areas (Li et al., 2009). (*Line: 70-82*)

**Lines 77-79 could use specificity and citations.**

- Response: Citations have been correctly added in the revised manuscript. (Line: 90)

**Line 81-83: Claims like this are made at various places in the manuscript. Sometimes (like here), there seems to be an unsupported degree of agency assigned to the plant. Elsewhere (559-562), a similar statement reflects more of what I have seen supported in the literature, and is also more informative.**

- Response: This was our mistake during writing. The sentence has been changed to: ‘In general, the number of species of microbial communities in rhizospheres is lower than that in bulk soil.’ (*Line: 91-92*)

**Line 87: I don't understand: “core bacterial microbiomes closely associated with host plants dominate the degree of variability of the entire bacterial community across different niche”**

- Response: We thank the reviewer for raising this issue. We do agree that the phrase ‘core bacterial microbiome’ here is inappropriate. This is the first mention of this concept in this article; the concept has also been mentioned in Beckers et al. (2017) and Wang et al. (2019). In the two cited articles, the relevant analyses of the ‘core bacterial microbiome’ were highlighted. According to the articles, the ‘core bacterial group’ responds positively to different influencing factors, and in turn affects the bacterial community structure of different niches to varying degrees. However, none of these articles has clearly stated that the core bacterial group can control the degree of variation of the entire bacterial community structure of different niches. The previous overstatement was a consequence of our misunderstanding. We have therefore corrected it as follows: ‘Findings from previous studies have also indicated that the core bacterial microbiome, which is closely related to the host plant, responds positively to different influencing factors; this can affect the degree of variation of the bacterial community structure of different niches.’ (*Line: 95-98*)

We believe this statement can be a summary of the two articles. Furthermore, the two articles (by Beckers et al. (2017) and Wang at al. (2019)) focus on the ‘core bacterial microbiome’, which has raised our attention. We are therefore interested in exploring the core bacterial microbiome composition at the microdomain of desert legume with an aim to provide supporting data on any future identification of dominant plant-related bacteria species and preparation of soil-improving bacterial agents. Therefore, in this study, the relevant analyses of the core bacterial microbiome were conducted at the position that is more important for all the analyses.

**Methods**

**Line 123: The sampling description is largely clear to the reader. Totals would enhance this clarity.**

- Response: We thank the reviewer for the comments and the overall evaluation of the description of the sampling.

**Line 130: Please describe somewhere in this section how tools were cleaned/sterilized across samples.**

- Response: The descriptions of the cleaning and disinfection of related sample collection tools have been revised as follows: ‘All sampling tools (tweezers, blades, etc.) were wiped and sterilized with alcohol (75%) after each use to prevent contamination of the samples from foreign bacteria.’ (*Line: 159-151*)

**Line 131: For bulk soil samples, is this from a zone where there are no roots? If not, were there species of non-focal shrubs nearby, and if so, is there anything the authors can report on how this varied or did not vary across sampling locations?**

- Response: With regard to the collection of the inter-shrub bulk soil, it took place at the exposed land (in which there is no distribution of roots) between the leguminous plants involved in this study, and the sampling depth was the same as the depth of root zone soil (10-40 cm). Since the entire sampling site was a natural enclosure, it is unavoidable that other plant species that are not involved in this study can also grow in the site. The relevant information has been incorporated into *line 134-137*: Since the sampling site was a natural enclosure, plants such as *Artemisia ordosica*, *Setaria viridis*, and *Salix psammophila* were sparsely distributed in the site. Thus, in this study, the interference of these plants (> 5 m away) was avoided during sampling.

**Line 137: Not sure what** **“interval” means in this context.**

- Response: We may have used an improper word ‘interval’ here. We intended to say: ‘The new PBS buffer was replaced during each repetition, and the time interval between the two operations was 5 minutes.’ We have changed the sentence in the revised manuscript accordingly. (*Line: 154-155*)

**Line 155: I infer roots are not analyzed for physicochemical properties, but the reader could use this explicitly stated.**

- Response: We thank the reviewer for this tip, which is indeed our own omission. The sentence has now been revised as follows: ‘Although the physical and chemical properties of root tissue were not measured, the subsequent analysis showed that rhizosphere soil associated endophytic bacteria communities were most closely interact with the physical and chemical properties of the rhizosphere soil, which is an indication that the two are correlated.’(*Line: 191-194*)

**Line 168: What restrictions? After reading the results, I think it makes more sense to simply drop the root samples and associated sequences from the analysis of soil physicochemical properties? The authors may disagree with me on this point.**

- Response: This part was amended as follows: ‘The portable soil moisture meter that was used at the time has a probe that is too large to accurately detect the soil water content (SWC) in a very small area of rhizosphere. In addition, the rhizosphere soil that was subsequently cleaned and separated by PBS buffer could not suitably be used for the measurement of soil water content, as the results may not be accurate. Therefore, the measured value of the soil in the root zone was only used as the soil moisture content shared by the rhizosphere and the root zone in the subsequent analysis and calculation.’ (*Line: 185-191*)

**Line 181: The selection of primers makes sense as a practical decision. However, as it isn’t considered best for capturing diversity of bacteria, the authors should add some discussion of how this could/should not have affected results.**

- Response: Regarding the lack of description here, we have revised the description by adding relevant citations, as well as comparing the feasibility of the citations. We have amended the sentences as follows: ‘Some studies have shown that using the variable regions V5–V7 (Zgadzaj et al., 2016; Liu et al., 2017; Sánchez-López et al., 2018) as the sequenced samples can more effectively reduce host contamination compared to using the V3–V4 regions (Wang et al., 2017; Luo et al., 2017; Zhu et al., 2018).’ (*Line: 209-211*)

**Line 189: I note QIIME2 is available, and has better analytical tools (e.g. amplicon sequence variants vs operational taxonomic units). For this study, I think version 1.8 is fine --I just want to bring this to the authors' notice, in case they want to try it out.**

- Response: We greatly appreciate the reviewer for this suggestion. We will actively try it in our future research.

**Line 198: I don't know what “the sequence directions were adjusted” means.**

- Response: This was an improper description of the quality control of the sequencing process. The description has been modified to match the standard quality control procedure. The samples were distinguished by the barcodes and the primers that are complementary to both ends of the sequence, and the number of mismatches allowed by the barcode was 0. (*Line: 226-228*)

**Statistics analysis：**

**section: This needs way more detail. I won't go line by line at all. I will recommend the authors consider using UNIFRAC distance rather than Bray-Curtis.** **It could help overcome** **OTU clustering errors by downweighting the influence of similar sequences, and would distinguish this analysis from the PCA (unless this is a UNIFRAC-based PCA, as I think QIIME can do?).**

- Response: We have taken in account all the reviewer’s suggestions and updated the content in the ‘Statistical analysis’ section. We have tried our best to process the data (and have also tried to outsource the data processing to a third party); the relevant analyses were repeated according to the recommendations of the reviewer. We completely understand the reviewer’s concerns about the OTU clustering errors caused by using the Bray-Curtis distance. We have therefore followed the reviewer's advice and used the ANOSIM analysis based on UNIFRAC to obtain the ANOSIM numerical table (and prepared a new Fig. S1), as can be found in the revised manuscript. In addition, the previous PCA analysis based on Euclidean Distance was replaced with PCoA based on UNIFRAC distance. For this, we have also consulted our partner—Beijing Allwegene Technology Co., Ltd., China, http://www.allwegene.com), and they have advised that the PCoA based on UNIFRAC distance can produce better results. However, we have decided to keep the basis of Bray-Curtis distance matrix based hierarchical clustering analysis (Fig. 2B), mainly because this analysis is the statistical calculation at the Phyla level, and Beckers et al. (2017) also performed their clustering analysis based on Bray–Curtis distance calculation. (*Line: 256-263*)

**Results:**

**This is not a complete line-by-line for this section, since I assume much text will change, but I have a few thoughts. I generally have not commented on the figures, as I expect they may change or that revised methods/results text might make them easier to interpret.**

**It is normal to report various stats on sequencing: e.g. total reads or bp sequenced, total reads post quality filters, range of read-depth per sample, # of OTUs, # of OTUs assigned taxonomy identifiers etc. etc.**

**Figure 1: I do not think the rarefaction curves look flat – i.e. not saturated. Did the authors use a method to estimated the number of OTUs they might have seen at saturation? I think they may have, but that descriptive detail is lacking? I also do not see referenced sequencing depth statistics (mean and SE). I think some text in the caption or following text includes an error: Good's coverage is non-overlapping, which instead suggests that some groups of samples are not sequenced as completely as others, but the conclusion is opposite.**

- Response: We agree with the reviewers 1 and 4’s opinions on this and have redrawn the Figure 1 according to the method of Beckers et al. (2017). As commented by Reviewer 4, the rarefaction curve shown in Figure 1 was determined to be 20,000 (sequence number, X-axis), and the corresponding line was not fully saturated. Therefore, in the newly drawn Figure 1, we have updated the description of the relevant analysis. We greatly appreciate the reviewer for such valuable and rigorous suggestions on revision; this is a very serious error. We have now re-analyzed the relevant data and hope to better describe the results on the actual rarefaction curve analysis. It has now been amended as follows: ‘For most root samples, when the sequence number was close to 20,000 (X-axis) and the corresponding value range was 1000-1500 OTUs (Y-axis), the curve reached saturation (Fig. 1A). For the under-shrub rhizosphere samples, the rarefaction curve reached saturation when the sequence number was close to 20,000 (X-axis) and the corresponding value range was 1500-3500 OTUs (Y-axis) (Fig. 1B). For the root zone soil samples, the rarefaction curve reached saturation when the sequence number was close to 25,000 (X-axis) and the corresponding value range was 1500-3500 OTUs (Y-axis) (Fig. 1C). For the bulk soil samples, the rarefaction curve reached saturation when the sequence number was close to 42,000 (X-axis) and the corresponding value range was 2000-4000 OTUs (Y-axis) (Fig. 1D).’ (*Line: 276-292*)

We have also removed the improper description of ‘Good’s coverage’. (Line: 290, 294-295)

**The phrasing in lines 272-273 seems out of sync with reported numbers in Table 1. The words suggest alphaRS>alphaB (not supported by data), but I think the authors mean to say: alphaB>alphaRZ, similar to how alphaRS>alphaRZ (supported by data).**

- Response: Regarding the analysis error in Table 1, which is similar to the comment from reviewer 1, we have changed the content in lines 271-273 to: ‘for the two *Hdysarum* species, the number of OTU, as well as their Chao1 and Shannon values of the samples collected in the rhizosphere soil were significantly higher than those of samples collected in the root and soil samples in the root zone (*P <* 0.05).’ (*Line: 310-312*)

**Line 282: “and exploring the main influencing factors driving differences in micro-community compositions”. PCA describes a pattern, not causality. Also on the PCA, why only report 2 axes? Do other axes not distinguish other groups (e.g. sites, species) or do they not explain sufficient variance?**

- Response: We thank the reviewer for this important comment. Since the main purpose of this article was to study the niche differentiation of rhizocompartments, and in the previous PCA analysis, the focus of the design of groups was to verify the differences between the bacterial communities of the four rhizocompartments. Only two axes with the largest components were reported, and the location and species of other axes were not fully considered when grouping the data.

According to the reviewer’s comment: ‘recommend the authors to do the PCA based on the UNIFRAC distance instead of the Bray-Curtis distance’, although we initially performed our PCA based on Euclidean Distance, after group discussion and consulting with relevant experts, we have now considered that doing the PCoA based on UNIFRAC distance would be a better idea. Thus, we have now used the PCoA1 and PCoA2 (Line: 328), which are the two largest principal coordinate values, to explain the structure of the bacterial communities; the two-dimensional figures select two coordinate axes that could better explain the influence of the sampling design on the rhizocompartments composition.

Regarding the erroneous description that was pointed out by the reviewer, we have deleted this description.

**Figure S1: There is no** **y-axis. Is it Bray-curtis distance? Why are bulk samples separated in panel a? No mention is made of the logic for the different test groupings in the methods.**

- Response: We have checked Fig. S1 and added y-axis. This is indeed ANOSIM analysis based on the Bray-Curtis Distance. We completely understand the concern of the reviewers’ concern regarding the Bray-Curtis Distance-based OTU clustering; thus, we have followed the reviewers' advice and performed the UNIFRAC-based ANOSIM analysis. The results of this analysis can be found in the ANOSIM Table 2 and Fig. S1 in the revised manuscript.

| Table 2 Analysis of similarity (ANOSIM) | | | | | | |  |  |
| --- | --- | --- | --- | --- | --- | --- | --- | --- |
| Phylogenetic level |  | Phylum | |  | OTU | | |  |
| ANOSIM output |  | *r* | *P-value* |  | *r* | *P-value* | |  |
| R vs. RS |  | 0.9499 | 0.001 |  | 0.9057 | 0.001 | |  |
| R vs. RZ |  | 0.9952 | 0.001 |  | 0.9585 | 0.001 | |  |
| RS vs. RZ |  | 0.4366 | 0.004 |  | 0.2541 | 0.023 | |  |
| R vs. Bulk |  | 1 | 0.001 |  | 0.987 | 0.001 | |  |
| RS vs. Bulk |  | 0.749 | 0.001 |  | 0.4784 | 0.001 | |  |
| RZ vs. Bulk |  | 0.0706 | 0.137 |  | 0.1022 | 0.08 | |  |
| Rhizocompartments |  | 0.6426 | 0.001 |  | 0.4953 | 0.001 | |  |
| Plant rhizocompartments effects on the microbial community structures were calculated using analysis of similarities (ANOSIM) based on the Weighted UniFrac distance metric (999 permutations).  R: root; RS: rhizosphere soil; RZ: root zone soil; Bulk: inter-shrub bulk soil. Significance levels: *P* ≤ 0.05; *P* ≤ 0.01. | | | | | | |  |  |
|  |  |  |  |  |  |  |  | |
|  |  |  |  |  |  |  |  | |
|  |  |  |  |  |  |  |  | |

In order to avoid the interference between Figure A and Figure B in Fig. S1, this new ANOSIM figure focuses only on the comparison of the four proposed type of rhizocompartments, and the original settings of image A was removed.

**Just in case: In plotting & analysis software, sometimes multiple “unknown” taxa from QIIME may be grouped together because they share the name “unknown” and not because they are the same OTU or even similar enough to be in the same order/phyla. They looked grouped in figures, because there is a very high abundance taxa with unknown identity (unusual but not impossible). This could be intentional for figures, but for analyses it would not make sense to group different unknown taxa into one taxa.**

- Response: Such rigorous and valuable tips help us to not only correct and improve our analyses, but also clarify the confusion during the preparation of figures. We are very thankful for these tips as we have benefited so much from them.

**Line 359: I do not know what “we used the OTU number of inter-shrub bulk soil as a control” means in this analysis.**

- Response: We set this control group according to the analysis method of Xiao et al. (2017), in which they performed verification and filter of the enrichment effect. According to their analysis, they believed that the root endophyte community in the root system of plants was mainly enriched with the bacteria in inter-shrub bulk soils through the rhizocompartments. In this study, we employed the same approach to investigate the enrichment of different microdomains in the root of desert legumes. The inter-shrub bulk soil was considered as the most distant niche of the four root microdomains and was least affected by the plants; thus, it was used as a control group in subsequent analyses.

**Line 361: The related figure (5) seems to only compare rhizocompartments, not OTUs? I don't understand.**

- Response: We thank the reviewer for raising this point. Regarding the method and analysis related to Figure 5, the reviewer recommended in the ‘Major Comments’ that the Metastates analysis of the significant difference between the communities may not be applicable to this study. We have carefully contemplated on this issue. After consulting with a third-party, a biological technology company Beijing Allwegene Technology Co., Ltd., China (http://www.allwegene.com), we have applied the Benjamini and Hochberg’s algorithm (Xiao at el., 2017) to analyse the OTU data and finally obtained a list of OTUs, of which R, RS, and RZ were significantly enriched or reduced (*P* ≤ 0.05) (S2 Table). In accordance with the list, we have then prepared a new Venn diagram. All new relevant analyses and description have been incorporated into the revised manuscript. (*Line: 403-419*)

**Figure 5: I don’t understand the Venn diagram figure or the analysis underlying it. How can 80 OTUs from the rootzone be enriched relative to the rootzone (in the root zone and bulk soil overlap)?**

- Response: Based on the problem raised by the reviewer, we have re-prepared the data and discussed them by referring to the method of Xiao et al. (2017). Since the Venn diagram shown in Figure 5 was generated using only one control group, whereas the other three groups were used for comparison, only two groups of OTUs that were significantly different were listed. In other words, each of the R, RS, and RZ groups of the OTU data was compared with the Bulk control group OTU. The diagram summarises and lists the groups that were significantly different; the obtained statistical differences between the OTU groups are also shown in the diagram to clearly show the overlaps of groups that had the same reduction or enrichment tendency.

**Line 417: What** **species indicator analysis? I didn't see this mentioned anywhere else...**

- Response: Our indicator species analysis was carried out based on the method of Beckers et al. (2017) and was outsourced to a third-party, the Beijing Allwegene Technology Co., Ltd., China (http://www.allwegene.com). The primary goal of the analysis was to further explore the unidentified effects of bacterial community differentiation on rhizocompartments, to add to the dominant effect of the core bacterial microbiomes. (*Line: 260-261, 442-446*)

**Table 2: what aspect of communities are soil properties correlated with? I couldn’t see it in the methods.**

- Response: Regarding this point, we have the description in line 295 in the Method section, which states that the Mantel test was used to determine and quantify the eight soil factors that could significantly affect the changes of the bacterial community structure in the rhizocompartments. In the discussion section, we have also specifically discussed the factors that affect the changes of the bacterial community structure in the root microdomains and referred to some of the explanation described in Sun et al. (2017).

**Discussion: This is not a careful line by line commenting.**

**The first two paragraphs here are largely conclusions and relevant literature, but no references to the specific results that support conclusions. Instead, the third paragraph is a list of relevant tests without specifics. See my major comment for where I think the authors did a much better job and compare the two.**

**Line 468: “Our results indicate that variations in specific core bacterial microbiomes in massive endophytes colonizing roots of various plant species” I do not understand this part, and so do not understand the whole sentence.**

- Response: This sentence in the original manuscript was unclear, and we have changed it in the revised manuscript as follows: ‘Our data showed that the uneven migration and distribution of the specific core bacterial microbiomes that are colonized in the root system of each plant species among the rhizocompartments are the main reason for the high variation of the rarefaction curves.’ (*Line: 484-486*)

**Lines 470-473: Do you have results supporting this claim? I did not see or understand them if so.**

- Response: This statement was quoted from the literature to mainly explain the reason for the revision of *Line 484-486*, as per your comment. It explains the reason for the uneven migration and distribution of specific core bacterial microbial groups in the rhizocompartments. Although the statement was deficient, and there were no supporting materials or results to support it, it was only to discuss and explain the previous statement. (*Line: 486-490*)

**Line 479: “must” is not a good word here, and elsewhere. Not all endophytes promote growth.**

- Response: We thank the reviewer for raising this point. We have amended the sentences as follows: ‘Bacteria that enter roots not only need to adapt to stress factors caused by the innate immune system of host plants, but also have complex interactions with host plants to promote plant growth.’ (*Line: 495-490*)

**Below illustrates my confusion on “structural variability”:**

**Lines 490- 491: “In summary, the colonization of endophytes in roots and the subsequent formation of a community with a relatively stable structural composition seem to constitute a highly variable process.” If the structure is stable, then where is the evidence for variability?**

- Response: The concept of ‘structural variability’ was adopted from Beckers et al. (2017). However, due to our poor analysis and description, we failed to fully demonstrate the concept in our original manuscript. However, we have carefully considered the reviewers’ advices and tried our best to make up for this shortcoming in this revised manuscript. We have prepared a new alpha rarefaction curve (Figure 1), and performed PCoA based on the UNIFRAC distance, and the similarity analysis of ANOSIM bacterial community structure, and the data can be found in Table 2. We do hope that the revisions are satisfactory and can ease the confusion of the reviewers.

**Line 510: Typo in order of treatments?**

- Response: Since Bacteroidetes in the soil root zone and in the inter-shrub bulk soil were not significantly different, the order of root microdomains was correct (no bulk soil). However, after proofreading the manuscript, we have been amended this part as follows: ‘Our results indicated that the relative abundance of Proteobacteria and Bacteroidetes in *C. microphylla* and *H. scoparium* shrubs could be ranked in the following order: root zone soil < rhizosphere < inside roots (*P* < 0.01). Additionally, the relative abundance of *Actinobacteria* in the shrub soil was significantly reduced compared to that of *Actinobacteria* in the roots (*P* < 0.05).’ (*Line: 529-533*)

**516-519: This text implies *Sphingobacteriia* is the only copiotrophic member of Bacteroidetes. It would surprise me if that were the conclusion of Fierer et al. I’m wondering if that’s truly what’s meant?**

- Response: Regarding this issue, we have read the relevant literature and found this information from two articles. Fierer et al. wrote under the heading ‘Copiotrophic and oligotrophic phyla’: ‘In contrast to the oligotrophic Acidobacteria, the beta-Proteobacteria, and Bacteroidetes exhibited copiotrophic attributes; their relative abundances were highest in soils with high C availability either a as an intrinsic property of the soil or as a result of sucrose amendments.’ Sun et al. cited the result of Fierer et al. in the discussion section of their paper, and in the second last paragraph, they wrote: ‘However, although Bacteroidetes (mostly Sphingobacteriia) exhibit copiotrophic attributes (Fierer et al. 2007)’. Unfortunately, we could not verify the accuracy of the quotation by Sun et al.; thus, we directly quoted the text from them. This leads to an inaccurate description of the eutrophication (copiotrophic attributes) of Bacteroidetes and *Sphingobacteriia*. The following sentence was added to the revised manuscript: ‘In addition, Fierer et al. (2007) have confirmed that, compared to the oligotrophic of Acidobacteria and Beta-Proteobacteria in soils under shrubs, Bacteroidetes have better symbiotic nutritional properties, …’. We hope that the reviewer satisfies with our reply and correction. (*Line: 539-541*)

**521-523: This is a bit of a cliff-hanger. Does pH explain the trend of Actinobacteria?**

- Response: These descriptions of the citations were in fact inaccurate. We have amended it as follows: ‘Research has found that the relative abundance of Actinobacteria is mainly affected by soil pH.’ (*Line: 545-546*)

**Line 525: What does “degree of significance in roots” mean? – (that’s how I read this.)**

- Response: This was our mistake, and we apologize for that. It has now been amended to: ‘Burkholderiales were significantly enriched in the roots.’ (*Line: 548*)

**Line 528: Why mention of aromatic compounds? Are they relevant? The reason is missing if so.**

- Response: The intent of this description was to show that Burkholderiales not only help in plant growth, but also have other functions. However, as the reviewer considered that this reference is not related to the content of this paper, and we have agreed to it. We have accordingly proceed to delete this content.

**Line 546-548: I am struggling to understand how this last sentence links in with Rhizobium & the data from the present study.**

- Response: We agree that the quotation here is inappropriate. However, we find that it is good for summarizing and verifying the content in lines 522-549, and thus have moved it to after *line 550* of the revised manuscript.

**Paragraph starting on 549: I think this paragraph was meant to discuss how soil/niche properties might explain which microbes occur in which rhizocompartments. There are results that speak to this, but this paragraph doesn’t clearly reference those results and is instead repetitive of the previous two paragraphs.**

- Response: We have updated the discussion on the physical and chemical properties of rhizocompartment soils and the Mantel Test results, as well as the RDA analysis. We have also provided new examples to the discussion on the specific core bacteria microbiome that is affected by main driving factors particularly in the rhizocompartment, to ensure that the discussion in this paragraph is clearer.

The newly added content is as follows: ‘For example, the core bacterial microbiomes *Bosea*, *Rhizobium*, and *Mesorhizobium*, which are abundant in the rhizosphere soil, are mainly affected by NO_3_^-^ and TN (Table 3 and Figures 6 and 7). Research shows that only a few microorganisms can absorb and assimilate nitrate nitrogen in soil. When nitrate nitrogen and ammonium nitrogen are present at the same time, ammonium nitrogen can inhibit the nitrate nitrogen absorption of microorganisms, because the assimilation of nitrate nitrogen requires energy consumption (Wickramasinghe et al. 1985). From the rhizocompartment to the root system, the interaction between plant roots and soil microorganisms is gradually strengthened, but the absorption preference of nitrogen-fixing microorganisms for ammonium nitrogen resulted in fixation and accumulated of ammonium nitrogen (Jenkinson et al. 1989; Recous et al. 1990). This results in the ammonium nitrogen content of rhizosphere soil to become significantly lower than nitrate nitrogen content (Table S5). Therefore, nitrogen in rhizosphere soil (NO_3_^-^ and TN) more markedly affects certain bacterial microbiomes (especially for *Bosea* and *Mesorhizobium*) (Table 3 and Figure 6).’ (*Line: 575-587*)

**562-564: Is this from the results (if so, which result specifically?) Or is this meaning to reference other work?**

- Response: We thank the reviewer for this valuable comment. This was an improper description of the literature to sum up the above discussion, it has been deleted from the manuscript.

**Line 570: I think the authors mean to say here that members of core microbiome of the root endophyte zone had a hierarchical enrichment. I think there is evidence for this. But as written this conclusion is confusing.**

- Response: We thank the reviewer for this valuable comment. The original description was not rigorous and accurate; thus, it has been deleted from the manuscript.

**Line 573-574: The first clause I understand and see how the results support. I cannot say the same for the second clause.**

- Response: Since the original analyses were not rigorous and accurate, they cannot support this conclusion. However, we do hope that some of the results in the revised manuscript (including the redrawn alpha rarefaction curve (Figure 1), the ANOSIM analyses of the bacterial community structure at both the taxonomic and OTU levels (Table 2), and the supplementary figure showing the ANOSIM analysis results) can support our conclusion.

**Line 577: “each rhizocompartment represented a unique niche of bacterial communities”. Unless I missed something, this is not the case. Instead, the PCA and Bray-Curtis results suggest all but endophytic communities are similar.**

- Response: We do agree that this statement is indeed an overstatement. Thus, the statement and other inappropriate conclusions have been deleted from the manuscript.

**Line 578-580. Clause 1 is supported by the data. For clause 2, I do not understand what the authors mean, exactly, and this is the first mention we've had in the discussion about restoration.**

- Response: Our description here was indeed inappropriate. Therefore, in the revised manuscript, we have changed it to: ‘This study provided data to support the identification of plant-related dominant bacteria and the preparation of soil improvement inocula in the future.’ Because this article provides and shows the results on the core bacterial groups of specific plants, it can be valuable for various future applications. Lastly, the original mention of ‘evaluation reference’ function was inappropriate. (*Line: 607-608*)

**Thanks again for the reviewer’s help to improve the manuscript.**

**Reference:**

Alguacil, M.M., Caravaca, F., Azcón, R., Pera, J., Díaz, G., Roldán, A., 2003. Improvements in soil quality and performance of mycorrhizal *Cistus albidus* L. seedlings resulting from addition of microbially treated sugar beet residue to a degraded semiarid Mediterranean soil. Soil Use Manag. 19, 277e283. <https://doi.org/10.1111/j.1475-2743.2003.tb00316.x>

Azcon, R., Medina, A., Roldan, A., Biro, B., Vivas, A., 2009. Significance of treated agrowaste residue and autochthonous inoculates (Arbuscular mycorrhizal fungi and *Bacillus cereus*) on bacterial community structure and phytoextraction to remediate soils contaminated with heavy metals. Chemosphere 75, 327e334. <https://doi.org/10.1016/j.chemosphere.2008.12.029>

Bashan, Y., Holguin, G., de-Bashan, L.E., 2004. *Azospirillum*-plant relationships: physiological, molecular, agricultural, and environmental advances (1997-2003). Can. J. Microbiol. 50, 521e577. <https://doi.org/10.1139/w04-035>

Beckers B, Op De Beeck M, Weyens N, Boerjan W, Vangronsveld J. Structural variability and niche differentiation in the rhizosphere and endosphere bacterial microbiome of field-grown poplar trees. Microbiome. 2017; 5: 25. <https://doi.org/10.1186/s40168-017-0241-2>

Beckers B, Op De Beeck M, Weyens N, Boerjan W, Vangronsveld J. Structural variability and niche differentiation in the rhizosphere and endosphere bacterial microbiome of field-grown poplar trees. Microbiome. 2017; 5: 25. <https://doi.org/10.1186/s40168-017-0241-2>

Caravaca, F, Alguacil, MM, Azcón, R, Parladé, J, Torres, P, Roldán, A, 2005. Establishment of two ectomycorrhizal shrub species in a semiarid site after in situ amendment with sugar beet, rock phosphate, and *Aspergillus niger*. Microb. Ecol. 49, 73e82. <https://doi.org/10.1007/s00248-003-0131-y>

Caravaca, F., Barea, J.M., Roldán, A., 2002. Synergistic influence of an arbuscular mycorrhizal fungus and organic amendment on *Pistacia lentiscus* L. seedlings afforested in a degraded semiarid soil. Soil Biol. Biochem. 34, 1139-1145 <https://doi.org/10.1016/S0038-0717(02)00047-0>

Caravaca, F., Figueroa, D., Roldán, A., Azcón-Aguilar, C., 2003. Alteration in rhizosphere soil properties of afforested *Rhamnus lycioides* seedlings in short-term response to mycorrhizal inoculation with Glomus intraradices and organic amendment. Environ. Manag. 31, 412e420. <https://doi.org/10.1007/s00267-002-2879-0>

Gao, L. F., Hu, Z. A., & Wang, H. X., 2002. Genetic diversity of rhizobia isolated from Caragana intermedia in Maowusu sandland, north of China. Letters in Applied Microbiology, 35(4), 347–352. <https://doi.org/10.1046/j.1472-765x.2002.01192.x>

Giraud, E., Moulin, L., V allenet, D., Barbe, V., Cytryn, E., Avarre,J. C., et al. (2007). Legumes symbioses: absence of *Nod* genes in photosynthetic bradyrhizobia. Science 316, 1307–1312. <https://doi.org/10.1126/science.1139548>

Jenkinson DS, Parry LC (1989) The nitrogen cycle in the broadbalk wheat experiment a model for the turnover of nitrogen through the soil microbial biomass. Soil Biology & Biochemistry 21(4): 535-542. <https://doi.org/10.1016/0038-0717(89)90127-2>

Li Lu, Y., Chen, W. F., Li Han, L., Wang, E. T., & Chen, W. X. (2009). Rhizobium alkalisoli sp. nov., isolated from Caragana intermedia growing in saline-alkaline soils in the north of China.Int J Syst Evol Microbiol. 59 (12): 3006-11. <https://doi.org/10.1099/ijs.0.007237-0>

Luo, G., Rensing, C., Chen, H., Liu, M., Wang, M., Guo, S., Ling, N., Shen, Q. (2018). Deciphering the associations between soil microbial diversity and ecosystem multifunctionality driven by long-term fertilization management. Functional Ecology, 32(4), 1103–1116. <https://doi.org/10.1111/1365-2435.13039>

Maron, J.L.; Marler, M.; Klironomos, J.N.; Cleveland, C.C. Soil fungal pathogens and the relationship between plant diversity and productivity. Ecol Lett. 2011, 14:36–41. <https://doi.org/10.1111/j.1461-0248.2010.01547.x>

Medina, A., Vassileva, M., Caravaca, F., Roldán, A., Azcón, R., 2004. Improvement of soil characteristics and growth of *Dorycnium pentaphyllum* by amendment with agrowastes and inoculation with AM fungi and/or the yeast Yarowia lipolytica. Chemosphere 56, 449e456. <https://doi.org/10.1016/j.chemosphere.2004.04.003>

Recous S, Mary B, Faurie G (1990) Microbial immobilization of ammonium and nitrate in cultivated soils. Soil Biology & Biochemistry 22(7):913-922. <https://doi.org/10.1016/0038-0717(90)90129-N>

Sablok G, Rosselli R, Seeman T, van Velzen R, Polone E, Giacomini A, La Porta N, Geurts R, Muresu R and Squartini A. (2017) Draft Genome Sequence of the Nitrogen-Fixing *Rhizobium sullae* Type Strain IS123^T^ Focusing on the Key Genes for Symbiosis with its Host *Hedysarum coronarium* L. Front. Microbiol. 8:1348. <https://dx.doi.org/10.3389%2Ffmicb.2017.01348>

Sun Y, Zhang YQ, Feng W, Qin SG, Liu Z, Bai Y, Yan R, Fa KY, 2017. Effects of xeric shrubs on soil microbial communities in a desert in northern China. Plant Soil. 2017; 414: 281–294. <https://doi.org/10.1007/s11104-016-3111-y>

Wang J, Zhang T, Li L, Li J, Feng Y and Lu Q (2017) The Patterns and Drivers of Bacterial and Fungal β-Diversity in a Typical Dryland Ecosystem of Northwest China. Front. Microbiol. 8:2126. <https://doi.org/10.3389/fmicb.2017.02126>

Wang Y, Zhang W, Ding C, Zhang B, Huang Q, Huang R, Su X. 2019. Endophytic communities of transgenic poplar were determined by the environment and niche rather than by transgenic events. Front Microbiol. 2019; 10: 588. <https://doi.org/10.3389/fmicb.2019.00588>

Wang, D., Yang, S. M., Tang, F., and Zhu, H. Y. (2012). Symbiosis specificity in the legume - rhizobial mutualism. Cell. Microbiol. 14, 334–342. <https://doi.org/10.1111/j.1462-5822.2011.01736.x>

Wang, Y.B., Zhang, W.X., Ding, C.J., Zhang, B.Y., Huang, Q.J., Huang, R.F., Su, X.H. (2019). Endophytic Communities of Transgenic Poplar Were Determined by the Environment and Niche Rather Than by Transgenic Events. Front. Microbiol, 26. <https://doi.org/10.3389/fmicb.2019.00588>

Wickramasinghe KN, Rodgers GA, Jenkinson DS (1985) Nitrification in acid tea soils and a neutral grassland soil: Effects of nitrification inhibitors and inorganic salts. Soil Biology and Biochemistry 17(2):249— 252. https://doi.org/10.1016/0038-0717(85)90123-3

Yates, R., Howieson, J., De Meyer, S. E., Tian, R., Seshadri, R., Pati, A., Reeve, W. (2015). High-quality permanent draft genome sequence of Rhizobium sullae strain WSM1592; a Hedysarum coronarium microsymbiont from Sassari, Italy. Standards in Genomic Sciences, 10(1). <https://doi.org/10.1186/s40793-015-0020-2>

Young, J. P. W., Crossman, L., Johnston, A., Thomson, N., Ghazoui, Z., Hull, K., et al. (2006). The genome of Rhizobium leguminosarum has recognizable core and accessory components. Genome Biol. 7: R34. <https://doi.org/10.1186/gb-2006-7-4-r34>

Zhu, F., Zhu, C., Doyle, E., Liu, H., Zhou, D., Gao, J. (2018). Fate of di (2‑ethylhexyl) phthalate in different soils and associated bacterial community changes. Science of The Total Environment, 637-638, 460–469. <https://doi.org/10.1016/j.scitotenv.2018.05.055>
