# Supplementary material for: Diversity and structural differences of bacterial microbial communities in rhizocompartments of desert leguminous plants
Source: PLoS One. 2020 Dec 22;15(12):e0241057. doi: 10.1371/journal.pone.0241057 (PMC7755220; doi:10.1371/journal.pone.0241057)
Supplement: Attachment — Submitted filename: Response to Reviewers.docx [file pone.0241057.s008.docx]

**Responses to all the reviewers’ comments on the manuscript:**

**“****Diversity and Structural Variability of Bacterial Microbial Communities in Rhizocompartments of Desert Leguminous Plants”**

**(****PONE-D-20-03936R1)**

**Submitted to *PLOS ONE* for publication**

**August 13, 2020**

**Response to Comments of Reviewer #1**

**Manuscript number**: PONE-D-20-03936R1

**Author(s)**: Ziyuan Zhou, Guodong Ding*, Minghan Yu*, Guanglei Gao,Yingying He

**Title**: Diversity and Structural Variability of Bacterial Microbial Communities in Rhizocompartments of Desert Leguminous Plants.

**General Comments:**

**No futher comments.**

**Response:**

**Thanks again to Reviewer 1 for carefully reviewing this article. After the first round of revision since the initial submission, this article has significantly improved in quality.**

**Response to Comments of Reviewer #2**

**Manuscript number**: PONE-D-20-03936R1

**Author(s)**: Ziyuan Zhou, Guodong Ding*, Minghan Yu*, Guanglei Gao,Yingying He

**Title**: Diversity and Structural Variability of Bacterial Microbial Communities in Rhizocompartments of Desert Leguminous Plants.

**General Comments:**

**The manuscript has improved in this current version and addressed my concerns about data analysis and presentation. However, this reviewer still believes that the writing needs to be improved by a scientific editor. Since certain wordings are still unchanged, here are some examples from version 1 that could be improved, but still unchanged in version 2.**

**Response:**

We are very grateful to the reviewer for taking the time to read the manuscript, pointing out problems of crucial importance, and offering helpful comments and valuable opinions about how to improve the manuscript. In response to the comments proposed by the reviewer this time, we have made point-by-point revisions. Specific revisions can be found in the revised manuscript submitted. It is our sincere hope that the revisions will address the shortages of the version 2. The revisions are detailed below: **Specific suggestions:**

1. **L319 "display" should be "visualize"**

- Response: We agree with the reviewer’s advice and have replaced the inappropriate word. (*Line: 313*)

2. **L465 "divided" should be "separated" or "grouped"**

- Response: We agree with the reviewer’s advice and have replaced the inappropriate word. (Line: *446*)

3. **L478 "interval" should be "value"?**

- Response: We agree with the reviewer’s advice and have replaced the inappropriate word. (Line: *459*)

4. **In L492 and other places in the article: "migration" may not be appropriate in this context. The microbiomes are snap shots of what the compositions are. There is no data to show that bacteria moved as the term "migration" implies.**

- Response: We agree with the reviewer's comment and replaced or removed the inappropriate words. *Lines 464, 465, 470:* We have removed or replaced the word ‘migration’ with‘distribution’ or ‘composition’, because our analysis only revealed an uneven distribution of specific bacterial groups in the rhizocompartments. Although we have proved the filtering and enrichment mechanisms, our methods and results could not validate and support the uneven migration of specific bacterial groups among rhizocompartments.

5. **L500: "a few" is probably better as** **"plant-selected" or "specific"**

- Response: We agree with the reviewer’s advice and have replaced the inappropriate word into ‘plant-selected’. (Line: *475*)

6. **L528 "soya bean" should be "soybean"**

- Response: We agree with the reviewer’s advice and have replaced the inappropriate word. (Line: *501*)

7. **L529 "record" should be "****exhibit"**

- Response: As per Reviewer 4’s comment, the original sentence was replaced. (*Lines 502–504*)

8. **L543 "contrary" should be "opposite"**

- Response: We also removed this sentence because the original sentence was repeated in *lines: 502–504*.

9. **L554 "position" should be "location"**

- Response: We agree with the reviewer’s advice and have replaced the inappropriate word. (*Line: 523*)

10. **L558 "prominent" should be "significant"**

- Response: We agree with the reviewer’s advice and have replaced the inappropriate word. (*Line: 527*)

11. **In version 2, L23 "while" should be "Moreover"?**

- Response: We agree with the reviewer’s advice and have replaced the inappropriate word. (*Line: 24*)

12. **These are just some obvious wordings I can pick up readily since this reviewer does not have time to edit the paper. There are subtle differences between words when they both translated to be the same in another language. Yet the actual meanings are very different. Please be aware of this and have a scientific editor help your overall writing.**

- Response: After revising our manuscript according to the different reviewers’ comments, we had it checked and edited by a professional editor.

**Response to Comments of Reviewer #3**

**Manuscript number**: PONE-D-20-03936R1

**Author(s)**: Ziyuan Zhou, Guodong Ding*, Minghan Yu*, Guanglei Gao,Yingying He

**Title**: Diversity and Structural Variability of Bacterial Microbial Communities in Rhizocompartments of Desert Leguminous Plants.

**Minor comments:**

**Line 538: Bacteroidetes does not need Italic, Make sure it is consistent with rest of the phylum name.**

**Response:**

We thank Reviewer 3 for reviewing our manuscript since the initial submission. The reviewer’s comments have helped improve the quality of this article. We are grateful to the reviewer for his/her professional and constructive suggestions. In response to the minor comments by Reviewer 3, we have proofread the whole text and corrected the errors. *Line 509*

**Response to Comments of Reviewer #4**

**Manuscript number**: PONE-D-20-03936R1

**Author(s)**: Ziyuan Zhou, Guodong Ding*, Minghan Yu*, Guanglei Gao,Yingying He

**Title**: Diversity and Structural Variability of Bacterial Microbial Communities in Rhizocompartments of Desert Leguminous Plants.

**General Comments:**

**As I reviewed the manuscript previously, I have primarily evaluated changes and responses to my previous comments.**

**The authors of this manuscript have made a number of substantial and commendable improvements to their manuscript. The text changes to the introduction have better set up the study. The methods have improved in the clarity of text, the use of UNIFRAC distance, the better treatment of alpha diversity metrics, the use of Benjamini and Hochberg's algorithm to handle multiple testing issues (though this was only a small part of my original critique), and the use of the full community data for tests of community composition correlation to soil variables. Further, interpretation of alpha diversity metrics in the results has also improved, and several of the major issues I raised have been addressed or become more minor issues.**

**However, 2 major concerns I detailed on initial review (I was reviewer #4) were not addressed adequately, and one not at all. Importantly, this means that the discussion and conclusions still make statements that are not supported, and in one case a conclusion that was not even tested. This is why I marked "no" on question 2. A number of my minor comments were also not fully addressed. I think this manuscript requires more substantial revision. Generally, I strongly suggest that the authors focus on rephrasing their conclusions and discussion to fit what they have actually observed. The data as analyzed are useful, there is no need to over-interpret.
Response:**

Thank you very much for giving us an opportunity to revise our manuscript. We appreciate reviewer 4 for the constructive comments and suggestions on our manuscript. We have studied reviewers’ comments carefully. According to the reviewers’ detailed suggestions, we have made a careful revision on version 1. A marked-up copy of our manuscript that highlights changes made to the version 1, which we would like to submit for your kind consideration.

**Major Comments:**

**1) I still see a conclusion about how variable communities from certain sample types were WITHIN that type (“the formation of bacterial microbial communities in root was a higher variability process under the joint influence of rhizocompartment habitats and host plants”, line 601), but I still see no clear test of the elements of this and there are several issues. First, changes in communities correlated to proximity to the plant do not indicate agency of the plant or even causation (correlation is also misinterpreted as causation in the discussion of correlated changes in microbial communities and soil physicochemical properties, see below), Second, I struggle to see where “higher variability” comes from. If I can find any result that the conclusions might be derived from, it is figure S1. If “higher variability” is such a key result, the rationale and results should be presented in the main text. Yet, I don't think ANOSIM is designed for this test, and is particularly weak on this point. “The anosim function can confound the differences between groups and dispersion within groups and the results can be difficult to interpret.” (quoted from package `vegan' manual for R, see Warton et al. 2012, full citation below). Alternatively, a few places in the text lead me to believe that the authors may instead be misusing “highly variable process” to describe the opposite pattern – a deterministic pattern (e.g. see line 514). The other conclusions drawn from ANOSIM with respect to differences between groups of samples (e.g. roots vs bulk soil as reported in Table 3) appear generally sound to me, and they agree with other methods.**

- Response: We sincerely thank Reviewer 4 for carefully pointing out the shortcomings of our manuscript with detailed explanations, which let us know better the misunderstandings we have in terms of parts of analysis methods in our research. The citation of related literature has broadened our scope of applying related methods, and set good standards for professional application. Such suggestions helped improve our knowledge and manuscript’s quality. Regarding the inappropriate statements related to ‘highly variable process’. We have revised and proofread the entire manuscript and have ensured that the manuscript no longer contains such inappropriate statements. The responses to the individual comments of the reviewers and the modifications are detailed below:

*Title:* We agree with the reviewer’s advice and have revised the title to ‘Diversity and Structural Differences of Bacterial Microbial Communities in Rhizocompartments of Desert Leguminous Plants’. Since the findings of this study do not completely support the hierarchical variability of bacterial communities among rhizocompartments.

*Lines 10, 26:* We also modified the phrase ‘structural variability’ in the abstract to ‘structural difference’.

*Lines 20–22:* Similarly, in the abstract, the differences in bacterial microbial community structure among the rhizocompartments have been emphasized instead of variability. We have accordingly modified the sentence as follows: ‘The dominant bacterial microbiome responsible for the differences in microbial community structure and composition across different niches of desert leguminous plants mainly consisted of Proteobacteria, Actinobacteria, and Bacteroidetes.’

Line 30: Accordingly, the keyword ‘structural variability’ has been replaced by ‘structural difference’.

*Lines 275-278:* This paragraph compares the rarefaction curves of the bacterial microbial communities of the four rhizocompartments. We have removed the incorrect description ‘root samples recorded a higher variability in the shape of rarefaction curves’ from the previous version of the manuscript. We have modified the results as: ‘Moreover, compared to various under-shrub soil samples, root sample rarefaction curves recorded a loose distribution in the saturated amplitude range and soil sample rarefaction curves (especially rhizosphere soil samples) recorded a more concentrated distribution in the saturated amplitude range.’

*Line 457:* Subtitle modification. We agree with the reviewer’s advice and have replaced the inappropriate word into ‘structural difference’.

*Line 460:* We have replaced the phrase ‘high variability’ in the sentence to ‘the differences’.

*Lines 474–475:* We have removed this sentence: ‘Results from our study indicate that, compared to soil bacterial communities under desert leguminous plant shrubs, bacterial communities in roots have a higher structural variability (Table 3, Fig. 1and S1 Fig.).’ (*Lines 498–500* in the first round of review). Further, the discussion on the causes of structural differences below this sentence has been merged with the previous paragraph.

We also have removed these inappropriate sentences: *Lines 507–508* in the first round of review*:* ‘In summary, the colonization of endophytes in roots and the subsequent formation of a community with a relatively stable structural composition seem to constitute a highly variable process.’ *Lines 505–506* in the first round of review*:* ‘thus, the migration and colonization of certain soil bacteria in the root system are highly variable processes.’

*Lines 487–491:* ‘This observation is supported by the Alpha rarefaction curve (Fig. 1), PCoA and RDA results (Fig. 2 and 7), ANOVA on the relative abundances of major contributing bacterial taxa among rhizocompartments (S1 Table), and ANOSIM on the structures of bacterial communities in this study (S1 Fig.).’ This part has been modified to ‘The structural differences of bacterial microbes in the rhizocompartments are demonstrated by the Alpha rarefaction curve (Fig. 1), PCoA analysis and RDA analysis (Fig. 2 and 6), and ANOVA of the relative abundances of major bacterial taxa among rhizocompartments (S1 Table) and ANOSIM of the structures of bacterial microbes in this study (S1 Fig.).’

Revision to Conclusion:

Based on the major concern expressed by Reviewer 4 over the conclusion, we have removed the inappropriate sentences describing structural variability (*Lines 597–599* in the first round of review*:* ‘*The variability of the structural compositions of root endophyte microbiomes in leguminous plants was far higher than that of under-shrub soil bacterial microbial communities.*’). The conclusion has now been revised as follows: ‘The structure composition of root endophytic communities significantly differed from the bacterial communities in the other three rhizocompartments; the bacterial species abundance and microbial diversity of the root endophytic communities were significantly less than that of the bacterial communities in the other three rhizocompartments.’ (added in *Lines 569-572*) ‘The formation of the root endophytic communities was influenced by the rhizocompartment and plant species.’ (added in *Lines 574-575*)

**2) I made a previous comment about circular analyses, which relates to R1's comment about similar data being presented in multiple figures. This is still true about this manuscript. However, the authors respond that they replicated the analyses of another paper in order to provide direct comparisons (Beckers et al 2017 and Wang et al 2019). I understand that it can be useful to compare across studies, even when previous methodologies have underlying issues. I now instead recommend that the authors state this reasoning in the methods, and results. They should also provide statements that remind readers what can and cannot be inferred from changes in proportion data, and tone down their inferences. Specifically, despite decreasing in relative abundance, a particular bacterial taxonomic group might actually be increasing in total abundance. Also, when some groups are relatively higher in abundance, the logical requirement is that others will be less relatively abundant, but only one of them must change in absolute abundance to create this result in relative abundance. Changes cannot be assigned with certainty to particular taxa. Likewise, when selecting taxa that are most relatively abundant across samples that include more and less complex communities, taxa relatively enriched in less complex communities (here, roots) will therefore be more “relatively abundant” when pooling across groups. This is why first limiting to relatively abundant taxa across samples and then asking if relatively abundant taxa are enriched in roots is circular.**

- Response: With due respect to Reviewer 4’s comments on ‘circular analysis’ represented in Figure 6 (initial submission) and Fig. S2 (first revision), that we employed only the cartographic method used in the works by Beckers et al. (2017) and Wang et al. (2019) to determine the core bacterial microbiome. The underlying issues of this method (e.g., calculation of the relative abundance and absolute abundance of the filtered bacterial genera) have not been addressed. Since we still have no confidence to resolve this problem, the solution we made might not be satisfactory.

In the first round of review, Reviewer 1 commented: “**For Fig. 6/Table S2, why pick the top ten rather than choose OTUs to define as “dominant” based on their relative abundance (e.g. anything comprising over X% of the total OTUs for all four samples)? I am also not sure what meaningful new information is added in Fig. 6 that is not already provided in Fig. 5C-D, other than including a specific representative species name. I suggest moving this tree to the supplement and shortening the corresponding section of the results.**”. Reviewer 1 considered Figure 6 (‘circular analysis’ Fig. S2 in first revision) less significant. However, at that time we insisted on retaining the figure. Further, after this round of review, considering the valuable suggestion by Reviewer 4 also, we realized that this part of the manuscript is less relevant to this context. Therefore, we have removed the analysis, discussion, and Conclusion related to the core bacterial microbiome (circular analyses). Meanwhile, we have retained Figures 4 and 5, which show the filtered and enriched dominant bacterial groups, without affecting the integrity of this article. However, due to insufficient knowledge and lack of sufficient time, we have not found a proper solution to this problem. In addition, we have revised all related texts and improved the Results and Discussion. We hope that the reviewer understands.

Meanwhile, removal of the results on *Lines 430–446* in the first round of review*:* ‘The core bacterial microbiome in the rhizocompartments of desert leguminous plants’ affected the analysis of soil factors on the aforementioned ‘the core bacterial microbiome’ shown in Figure 6B in the section ‘Relationships between rhizocompartment bacterial microbial communities and soil factors’. Therefore, we used 20 dominant genera relatively enriched or depleted among the rhizocompartments (Fig. 5C and D) to analyse the effects of soil factors. Accordingly, few sentences have been revised as follows:

**Fig. 6** **Ordination plots of the results from the redundancy analysis (RDA) on the bacterial communities in rhizocompartments and soil factors.** (A) Effects of soil factors on the samples of four rhizocompartment types under three leguminous plant shrubs. (B) Effects of soil factors on the major contributing bacterial taxa (20 filtered dominant genera in Fig. 5C and D) in four rhizocompartments under three leguminous plant shrubs. CM: *Caragana microphylla*; HM: *Hedysarum mongolicum*; HS: *Hedysarum scoparium*. r: root; rs: rhizosphere soil; rz: root zone soil; b: inter-shrub bulk soil. Data of soil physicochemical factors are listed in S3 Table.

*Lines 19, 465, 546, 567, 574:* ‘the core bacterial microbiome’ has been replaced with ‘the specific bacterial microbiome’.

*Lines 22–24:* We have removed the summary on the core bacterial microbiome from the abstract and have added the results on soil factors: ‘All soil factors of rhizosphere and root zone soils, except for NO_3_^-^-N and TP under *C. microphylla* and the two *Hedysarum spp*., recorded significant differences (*P* < 0.05).’

*Lines 438-440:* We have modified this line to ‘Effects of soil factors on the major contributing bacterial taxa (20 filtered dominant genera in Fig. 5C and D) in four rhizocompartments under three leguminous plant shrubs’.

**3) Much data analysis text is still insufficient and still found in the results instead of the methods (for an example, see paragraph starting line 431, but this occurs elsewhere too). However, this problem has been reduced. Some paragraphs of the discussion still don't make sense (in combination with my first major point, this is largely why I marked "no" on question 5), however this problem is now lessened.**

- Response: We thank the reviewer for the detailed and valuable comments. We have verified our data analysis, removed and revised the parts questioned by the reviewers (previously and this time), and made necessary modifications in Discussion. Details are not listed here; please refer to the revised manuscript. We hope this revised manuscript has largely overcome these shortcomings.

**Other comments that were insufficiently addressed, or are based on edited material or a new understanding of previous material:**

**1) “Adaptability” which refers to the adaptive, evolutionary potiential of a population, is still used to refer to the concept that individuals may be able to tolerate a wider range of environments (in many places throughout). The authors should use “wide tolerance” or “plasticity” in most of these cases.**

- Response: We thank the reviewer for pointing out the errors in wording. We have made the necessary corrections, which helped improve the scientific explanation. Word revisions are as follows:

*Line 45:* ‘Adaptability’ has been replaced by ‘wide tolerance’.

*Line 58, 70, 127:* ‘Adaptability’ has been replaced by ‘plasticity’.

**2) My hologenome comment was mis-interpreted. I took issue with the intent, not the vocabulary. See, for example: Douglas, Angela E., and John H. Werren. "Holes in the hologenome: why host-microbe symbioses are not holobionts." MBio 7.2 (2016).**

- Response: We thank the reviewer for bringing up this point. The review (Douglas et al., 2016) suggested by the reviewer helped us gain some important information about ‘hologenome’ in the abstract: ‘A host plus its microbiome is more effectively viewed as an ecological community of organisms that encompasses a broad range of interactions (parasitic to mutualistic), patterns of transmission (horizontal to vertical), and levels of fidelity among partners. The hologenome requires high partner fidelity if it is to evolve as a unit. However, even when this is achieved by particular host-microbe pairs, it is unlikely to hold for the entire host microbiome, and therefore the community is unlikely to evolve as a hologenome.’ We have removed few sentences (*Lines 95–98* in the first round of review*:* ‘Findings from previous studies have also indicated that the core bacterial microbiome, which is closely related to the host plant, responds positively to different influencing factors; this can affect the degree of variation of the bacterial community structure of different niches.’) from our manuscript after reading the paper carefully. We once again thank the reviewer for the comment, which helped expand our knowledge and gave more ideas.

**3) I do not understand how the results on lines 284 -292 were generated. These curves still don't look like they are leveling off, although I agree with the authors that the method to draw them & relevant methods text seems improved. I suggest the authors acknowledge that diversity in any one sample might not be fully characterized due to sequencing depth – and again, tone down conclusions to fit this reality. Low power doesn’t make research useless, but it is very important to fully communicate the certainty of conclusions. Note this also relates to material in the results and discussion.**

- Response: Dear reviewer, the explanation about results was largely derived from graph-based comparative analysis. Rarefaction curve was plotted following the method used in similar studies. We agree with the reviewer's suggestion and have made the necessary revision as follows: ‘While the Good’s coverage of all rhizocompartment samples (roots, rhizosphere soil, root zone soil, and inter-shrub bulk soil) was comparable, the diversity was not fully characterized due to insufficient sequencing depth. In this study, Good’s coverage ranged from 95.3% to 96.9%; Good’s coverage of the soil samples was significantly less than that of the root samples (*P* < 0.05).’ *Lines 289–293*

We have also revised the explanation about the rarefaction curve in Results and Discussion to avoid overstatement in Discussion and Conclusion. *Line 459-462:* ‘Rarefaction curve indicates a correlation between the differences in species abundance (OTUs) among the rhizocompartments of desert leguminous plants and the enrichment and filtration of specific probiotic bacteria by roots in the under-shrub soil bacterial communities under different growth states of various sample plants.’

*Line 467:* As per the reviewer’s comment, we have tired our best to eliminate overstatement in conclusion and discussion by replacing ‘high variation’ with ‘the difference’.

**4) Table 1: The origin of letters denoting statistical differences are not fully explained, and therefore, some presented results are confusing. For example, it seems implied that in Shannon diversity, 9.10±0.07 b is different than 9.11±0.48 a, but I can easily see that standard errors overlap. So what are a and b telling me? This occurs at several places in the table. Conversely, bulk soil often appears to be not significantly less diverse than rhizosphere soil, so I struggle to believe the claim that there are significant “peaks” in diversity in the rhizosphere soil (as claimed on 309-310).**

- Response: We have also verified the alphabets used to denote the statistical differences in Table 1 and corrected the errors. The description of the significant peak of diversity in rhizosphere soil was not accurate, and therefore, we have removed the relevant sentence. (*Line 309* in the first round of review*:* ‘and that they all attained peaks in rhizosphere soil.’)

| **Table 1** High-throughput genome sequencing results of the rhizocompartment samples of the three leguminous plants. | | | | |
| --- | --- | --- | --- | --- |
| α-diversity index | | OTU | Chao1 | Shannon |
| Shrub species | Rhizocompartments |  |  |  |
| CM | r | 1086±63 b | 1682.23±32.65 b | 7.15±0.33 b |
|  | rs | 2300±224 a | 3244.72±306.22 a | 9.50±0.19 a |
|  | rz | 2260±99 a | 3192.19±242.44 a | 9.33±0.33 a |
|  | b | 2025.57±192 a | 3037.48±306.13 a | 9.33±0.22 a |
| HM | r | 1436±191 c | 2133.36±105.03 c | 7.34±0.99 c |
|  | rs | 2340±148 a | 3098.14±117.83 a | 9.53±0.20 a |
|  | rz | 2014±108 b | 2790.81±100.36 b | 9.08±0.05 b |
|  | b | 2059±192 ab | 2842.44±127.15 b | 9.16±0.48 ab |
| HS | r | 1319±126 c | 2101.61±143.51 c | 7.24±0.54 c |
|  | rs | 2374±161 a | 3309.04±130.52 a | 9.56±0.16 a |
|  | rz | 1998±92 b | 3050.33±81.11 b | 9.10±0.07 b |
|  | b | 2025.07±355 ab | 2863.53±150.19 b | 9.11±0.48 ab |
| *F*_shrub species_ | | 0.57 ns | 0.14 ns | 0.10 ns |
| *F*_rhizocompartments_ | | 47.72 | 30.64 | 71.05 |
| *F*_shrub species×rhizocompartments_ | | 7.70 | 8.08 | 5.47 |
| Data are presented as mean ± standard deviation, n=3. CM: *Caragana microphylla*; HM: *Hedysarum mongolicum*; HS: *Hedysarum scoparium*.  r: root; rs: rhizosphere soil; rz: root zone soil; b: inter-shrub bulk soil.  Different letters signify significant differences among four rhizocompartments (P < 0.05). ns: no significant difference.  The last three rows represent the F values of the interactions between plant species/rhizocompartment types (shrub species×rhizocompartments). | | | | |
|  |  |  |  |  |
|  |  |  |  |  |

**5) I note PCoA, ANOSIM, and hierarchical clustering give slightly different results, but this is not discussed.**

- Response: We have again verified the comment on supplementary discussion and explanation by Reviewer 4 during the first round of revision (**I will recommend the authors consider using UNIFRAC distance rather than Bray-Curtis. It could help overcome OTU clustering errors by downweighting the influence of similar sequences.**). We have revised it as follows: ‘We found that the results of PCoA, ANOSIM, and hierarchical clustering analysis were slightly different. PCoA and ANOSIM are methods based on UniFrac distance algorithm whereas hierarchical clustering analysis is based on Bray-Curtis distance algorithm. UniFrac distance algorithm could avoid errors in OTU clustering by reducing the influence of similar sequences.’ (*Lines 337–341*)

**6) I still do not understand why the analyses or comparisons in Figure 5 are undertaken. This may be related to my point about circularity of proportion data and the Becker paper.**

**Fig. 5 Venn diagrams of the significantly enriched (A) and depleted (B) OTUs in the other three rhizocompartments compared to root zone soil under three leguminous plant shrubs.** (C) Bacterial genera corresponding to relatively enriched OTUs; (D) Bacterial genera corresponding to relatively depleted OTUs. The enrichment and filtration effects of OTUs among rhizocompartments are listed in S2 Table.

- Response: Venn diagram analysis (Fig. 5) was initially performed using the methods described by Xiao et al. (2017). After the first revision, the analysis was reperformed using Benjamini and Hochberg's algorithm to determine the enriched or depleted OTUs of different rhizocompartments (the corresponding dominant genera). We did not employ the method by Beckers et al. (2017); however, the reviewer again raised concern during the second review. Although Figs. 5C and D also show the structural proportion of the relative abundance of the filtered specific genera the different rhizocompartment in the bacterial microbial communities in different rhizocompartments. In this data, we compare a series of values of composition proportion of bacterial microbial community structure among the rhizocompartments. We respect the reviewer’s viewpoint on ‘relative abundance’ and ‘absolute abundance’. However, due to the different analysis perspective, we believe that we can avoid Reviewer 4’s comment. ‘**This may be related to my point about the circularity of proportion data and the Becker paper.**’ Therefore, the revised manuscript emphasizes the proportion of specific bacteria in the bacterial community structure of the rhizocompartment rather than directly comparing the relative abundance or absolute abundance of specific bacteria genera in different rhizocompartments. By doing this, we conclude ‘relative’ filtering enrichment or depletion. We believe this eliminates the error caused by the uncertainty in the absolute abundance of specific bacterial genera in the bacterial microbial communities in different rhizocompartments. Several studies, including that by Sun et al. (2017) and Xiao et al. (2017), have compared the relative abundance of specific bacteria in microbial communities from different samples and plant tissues. We simply followed this approach in our current study too.

*Lines 417–419:* We performed statistical analysis for ten relatively enriched dominant bacterial genera corresponding to OTUs. The analysis revealed that these genera in their bacterial microbial community structure of the rhizocompartment were relatively enriched.

*Lines 426-427*: It shows that the proportion of these dominant bacterial genera in the rhizocompartment relatively depleted towards the roots.

**7) On line 487 the authors state about their results that “In fact, the enrichment of bacterial communities towards rhizosphere soil mainly derived from the following sources: (i) carbon-containing primary and secondary metabolites (root exudates) in plant rhizospheres; and (ii) allelochemicals released by plants through allelopathy, which induces chemotaxis in oil microbes [40]” but there is no experimental result documenting this, or method testing it. I recommend the authors remove this and similar conclusions that are unsupported.**

- Response: We agree with the reviewer’s comment and have removed this conclusion.

**8) Paragraph beginning 522:** **This paragraph is hard to parse. It may need to be simplified, especially given that relative enrichment and absolute abundance are not necessarily linked, see my major comments.**

- Response: We thank the reviewer for this insightful comment. We have revised the conclusions derived from the comparative analysis of ‘relative abundance and absolute abundance’ and no longer given ranks to the relative abundance of specific bacteria.

*Lines 502–505:* This part was amended as follows: ‘Results from our study indicate that a significant depletion in the relative abundance of Actinobacteria in bacterial microbial community structure in each rhizocompartment from the inter-shrub bulk soil to roots; Proteobacteria and Bacteroidetes in bacterial microbial community structure in each rhizocompartment showed enrichment towards the roots.’

We have deleted less significant results described *lines 542-544* in the first round of review: ‘However, Actinobacteria results in our study presented a contrary trend, recording a significant trend of depletion in the order of root<rhizosphere soil<root zone soil (*P* < 0.05).’

**9) Paragraph beginning line 553: This is useful content, but readers should be reminded that nodules were explicitly avoided in sampling, so the lack of enrichment of nodule taxa is somewhat unsurprising.**

- Response: We agree with the reviewer’s comment and have revised it in *lines 522–526* as follows: ‘No nodule samples were collected and analysed in this study; however, the degree of microbial diversity in the roots of desert leguminous plants is vast, the dominant positions of *Bradyrhizobium* and *Rhizobium* in the roots of *C. microphylla* and *H. mongolicum* are also predictable (Fig. 4 and 6, and S1 Table) as bacteria of these two genera are known to have symbiotic rhizobia, and they are closely related to the symbiotic nitrogen fixation of leguminous plants (Kaschuk et al., 2009; Xiao et al., 2017).’

**10) line 506: This language ascribes more agency to plants, than is actually supported by Bulgarelli. – likewise on 503, a better interpretation is that communities are not purely random. They could be deterministically opportunistic. I think this is a grammar error.**

- Response: Dear reviewer, as per your comment, we have verified the findings of Bulgarelli et al. (2012). We believe that our analysis reveals that soil type of the rhizocompartment determines the composition of bacterial microbial communities. Plant species plays a minor role here; however, that cannot be ignored. Therefore, we have modified the statement that host plant genes determine the karyotype distribution of bacterial microbial communities in rhizocompartments. It has been revised in *lines 480–482* as follows: ‘Bulgarelli et al. pointed out that the soil type determines the composition of bacterial communities in roots; however, the influence of host genotype on the karyotype distribution cannot be ignored (Bulgarelli et al. 2012).’

*Line 478:* We agree with the reviewer’s advice and have replaced the inappropriate word into ‘purely random’.

**Thanks again for the reviewer’s help to improve the manuscript.**

**Reference:**

Beckers B, Op De Beeck M, Weyens N, Boerjan W, Vangronsveld J. Structural variability and niche differentiation in the rhizosphere and endosphere bacterial microbiome of field-grown poplar trees. Microbiome. 2017; 5: 25. <https://doi.org/10.1186/s40168-017-0241-2>

Bulgarelli D, Rott M, Schlaeppi K, Ver Loren van TE, Ahmadinejad N, Assenza F, et al. Revealing structure and assembly cues for *Arabidopsis* root-inhabiting bacterial microbiota. Nature. 2012; 488: 91–5. <https://doi.org/10.1038/nature11336>

Douglas AE, Werren JH. Holes in the Hologenome: Why Host-Microbe Symbioses Are Not Holobionts. mBio, 2016; 7(2): e02099-15. <https://doi.org/10.1128/mBio.02099-15>

Kaschuk G, Kuyper TW, Leffelaar PA, Hungria M, Giller KE. Are the rates of photosynthesis stimulated by the carbon sink strength of rhizobial and arbuscular mycorrizal symbioses? Soil Biol Biochem. 2009; 41: 1233-1244. <https://doi.org/10.1016/j.soilbio.2009.03.005>

Sun Y, Zhang YQ, Feng W, Qin SG, Liu Z, Bai Y, Yan R, Fa KY, 2017. Effects of xeric shrubs on soil microbial communities in a desert in northern China. Plant Soil. 2017; 414: 281–294. <https://doi.org/10.1007/s11104-016-3111-y>

Wang Y, Zhang W, Ding C, Zhang B, Huang Q, Huang R, Su X. Endophytic communities of transgenic poplar were determined by the environment and niche rather than by transgenic events. Front Microbiol. 2019; 10: 588. <https://doi.org/10.3389/fmicb.2019.00588>

Xiao X, Chen W, Zong L, Yang J, Jiao S, Lin Y, Wang E, Wei G. Two cultivated legume plants reveal the enrichment process of the microbiome in the rhizocompartments. Mol Ecol. 2017; 26(6): 1641–1651. <https://doi.org/10.1111/mec.14027>
