# Supplementary material for: Diversity and structural differences of bacterial microbial communities in rhizocompartments of desert leguminous plants
Source: PLoS One. 2020 Dec 22;15(12):e0241057. doi: 10.1371/journal.pone.0241057 (PMC7755220; doi:10.1371/journal.pone.0241057)
Supplement: Attachment — Submitted filename: Response to Reviewers.docx [file pone.0241057.s009.docx]

**Responses to all the reviewers’ comments on the manuscript:**

**“****Diversity and Structural Variability of Bacterial Microbial Communities in Rhizocompartments of Desert Leguminous Plants”**

**(PONE-D-20-03936R2)**

**Submitted to *PLOS ONE* for publication**

**September 29, 2020**

**Response to Comments of Reviewer #2**

**Manuscript number**: PONE-D-20-03936R2

**Author(s)**: Ziyuan Zhou, Minghan Yu*, Guodong Ding*, Guanglei Gao,Yingying He

**Title**: Diversity and Structural Differences of Bacterial Microbial Communities in Rhizocompartments of Desert Leguminous Plants.

**General Comments:**

**No futher comments.**

**Response:**

Thanks again to Reviewer 2 for carefully reviewing this article. After the second round of revision since the initial submission, this article has significantly improved in quality.

**Response to Comments of Reviewer #4**

**Manuscript number**: PONE-D-20-03936R2

**Author(s)**: Ziyuan Zhou, Minghan Yu*, Guodong Ding*, Guanglei Gao,Yingying He

**Title**: Diversity and Structural Variability of Bacterial Microbial Communities in Rhizocompartments of Desert Leguminous Plants.

**General Comments:**

**The authors have made careful revisions that have improved the clarity and quality of the manuscript. I think the conclusions drawn from the data and analyses are now largely sound (with minor exceptions, see first point), and that the study represents a valuable contribution.**

**I was the original R4, and R4 the last time around. I write with respect to how my comments on the previous submitted version (revision 1) have been addressed in this version (revision 2). Every change I suggest now represents a minor change.**

**Response:**

We are grateful to Reviewer 4 for his/her rigorous and professional review of this manuscript. We thank Reviewer 4 for the useful and high-quality comments, which helped to improve the quality of the manuscript. More importantly, the scientific communication with the reviewers helped enhance our knowledge. We gained a deeper understanding of the use of relevant research methodologies in this field. We once again thank Reviewer 4 for the review from which we benefited a lot.

**Major Comments:**

**In response to my first major point, the authors have clarified text and results and I am largely satisfied with the response. Only this portion “correlation is also misinterpreted as causation in the discussion of correlated changes in microbial communities and soil physicochemical properties,” remains a point of concern to me. The following wording changes would be sufficient to correct it:**

**Line 437 “Effects of soil factors on the” should be “Correlation of soil factors to the”**

- Response: We agree with the reviewer's suggestion and have made revisions to some inappropriate statements. *Lines 440*

**Line 448 “****were mainly influenced by” should be “****were most strongly correlated to” – same for line 449, line 451, and line 453.**

- Response: We agree with the reviewer's suggestion and have made revisions to some inappropriate statements. *Lines 451, 452, 454-455*

**Line 539: “affecting” should be “****correlated with” and likewise Line 547: “affected by” should be “correlated with”.**

- Response: We agree with the reviewer's suggestion and have made revisions to some inappropriate statements. *Lines 542, 550*

**Line 554: “This results in” should be “****This probably explains our observation that”**

- Response: We agree with the reviewer's suggestion and have replaced the inappropriate phrase into“This probably explains our observation that”. *Lines 557*

**Line 556: “more markedly affects” should be “may differences in”**

- Response: We agree with the reviewer's suggestion and have made revisions to some inappropriate statements. *Lines 559*

**In response to my second major point – we are going to have to agree to disagree about what constitutes circularity in an analysis. However, the authors have removed all the conclusions that I thought were erroneous that hinged on the circular analysis. So I am satisfied.**

- Response: We thank Reviewer 4 for the helpful comments. We are glad that our previous point-by-point modification was satisfactory.

**In response to my third major point, I think both readability and distribution of methods text between methods/results have improved sufficiently.**

- Response: We are glad that our previous point-by-point modification was satisfactory. Thanks again for the reviewer’s help to improve the manuscript.

**In response to minor points**

1) **there are still a few instances of “adapt” that aren't right. I highlighted all of them below.**

- Response: We agree with the reviewer’s comments. We have made the necessary revisions for the inappropriate vocabulary.

**2 & 3) Good responses, I am satisfied with these changes.**

- Response: We thank Reviewer 4 for the helpful comments. We are glad that our previous point-by-point modification was satisfactory.

| **Table 1** High-throughput genome sequencing results of the rhizocompartment samples of the three leguminous plants. | | | | |
| --- | --- | --- | --- | --- |
| α-diversity index | | OTU | Chao1 | Shannon |
| Shrub species | Rhizocompartments |  |  |  |
| CM | r | 1086±63 b | 1682.23±32.65 b | 7.15±0.33 b |
|  | rs | 2300±224 a | 3244.72±306.22 a | 9.50±0.19 a |
|  | rz | 2260±99 a | 3192.19±242.44 a | 9.33±0.33 a |
|  | b | 2025.57±192 a | 3037.48±306.13 a | 9.33±0.22 a |
| HM | r | 1436±191 c | 2133.36±105.03 c | 7.34±0.99 c |
|  | rs | 2340±148 a | 3098.14±117.83 a | 9.53±0.20 a |
|  | rz | 2014±108 b | 2790.81±100.36 b | 9.08±0.05 b |
|  | b | 2059±192 ab | 2842.44±127.15 b | 9.16±0.48 ab |
| HS | r | 1319±126 c | 2101.61±143.51 c | 7.24±0.54 c |
|  | rs | 2374±161 a | 3309.04±130.52 a | 9.56±0.16 a |
|  | rz | 1998±92 b | 3050.33±81.11 b | 9.10±0.07 b |
|  | b | 2025.07±355 ab | 2863.53±150.19 b | 9.11±0.48 ab |
| *F*_shrub species_ | | 0.57 ns | 0.14 ns | 0.10 ns |
| *F*_rhizocompartments_ | | 47.72 | 30.64 | 71.05 |
| *F*_shrub species×rhizocompartments_ | | 7.70 | 8.08 | 5.47 |
| Data are presented as mean ± standard deviation, n=3. CM: *Caragana microphylla*; HM: *Hedysarum mongolicum*; HS: *Hedysarum scoparium*.  r: root; rs: rhizosphere soil; rz: root zone soil; b: inter-shrub bulk soil.  Different letters signify significant differences within species among four rhizocompartments (P < 0.05). ns: no significant difference.  The last three rows represent the F values of the interactions between plant species/rhizocompartment types (shrub species×rhizocompartments). | | | | |
|  |  |  |  |  |
|  |  |  |  |  |

**4) I am satisfied. It would (optionally) add even more clarity to change “Different letters signify significant differences among four rhizocompartments” in the Table 1 caption to “Different letters signify significant differences within species among four rhizocompartments”.**

- Response: We agree with the reviewer's suggestion and have made revisions to some inappropriate statements in Table 1.

**5) The changes are good.**

- Response: We thank Reviewer 4 for the helpful comments. We are glad that our previous point-by-point modification was satisfactory.

**6) Sorry, still don't understand panels A and B in Fig 5. Specifically, from reading the caption, I interpret that panels A and B show the number of taxa in the other samples that are enriched relative to root zone soil. Taxa in enriched in two other sample types, would then be summed in the overlap of corresponding colors. Yet numbers appear in zones that overlap with root zone sample. How can root zone be enriched relative to itself? i.e. in A, the overlap between rhizosphere, root zone, and bulk soil has the number 159.**

- Response: This is the wrong statement of our previous version, we give the wrong control measurement in the caption of Figure 5, which caused confusion to the reviewers' understanding. The wrong control measurement (the root zone soil) has replaced into “the inter-shrub bulk soil”. This part has been modified to “Fig. 5 Venn diagrams of the significantly enriched (A) and depleted (B) OTUs in the other three rhizocompartments under leguminous plant shrubs compared to the inter-shrub bulk soil.” *Lines 409-410*

**7)-10) no further comments, changes seem good.**

- Response: We thank Reviewer 4 for the helpful comments. We are glad that our previous point-by-point modification was satisfactory.

**Quick wording changes to make meaning appropriate/clear.**

**Line 42: “adaptable” should be “tolerant”**

- Response: We agree with the reviewer's suggestion and have revised the text. *Line 42*

**Line 82: “adaptations” should be “tolerance”**

- Response: We agree with the reviewer's suggestion and have made revisions to some inappropriate statements. *Line 82*

**Line 86: “differential variations” should be “differentiation”**

- Response: We agree with the reviewer's suggestion and have made revisions to some inappropriate statements. *Line 86*

**Line 96: same as line 86.**

- Response: We agree with the reviewer's suggestion and have made revisions to some inappropriate statements. *Line 96*

**Line 99: “variability” should be “differentiation”**

- Response: We agree with the reviewer's suggestion and have made revisions to some inappropriate statements. *Line 99*

**Line 104: I would delete the rest of this sentence after “rhizocompartments” as there is no test of it. However, the conclusions are fine, so leaving this as a hypothesis that remains untested may be what the authors prefer. I would leave this up to the authors.**

- Response: We agree with the reviewer's comment and have deleted the untested hypothesis pointed out by the reviewer. *Line 104*

**Line 127: “plasticity” should be “tolerance”**

- Response: We agree with the reviewer's suggestion and have made revisions to some inappropriate statements. *Line 126*

**Line 190: this phrase might be unclear “were most closely interact with the physical and chemical properties”. I think the authors mean that because the root communities are correlated to physicochemical soil properties near the root, it suggests that near-root soil properties might influence them. If I have interepreted this correctly than it should be fine to leave as is.**

- Response: We thank the reviewer for the comment. We believe that the reviewer’s understanding matches this sentence; we decided to leave it as it is. *Lines 189-190*

**Line 226: Should specify which databases for which sequencing data.**

- Response: We agree with the reviewer’s comment and have added the specific database information (Silva-SSU128-16S rRNA database-bacteria, https://www.arb-silva.de) used in the quality control of the sequencing data in this study. *Line 225*

**Table 3 appears inside the caption of Figure 6 (line 437).**

- ****Response: We thank the reviewer for their careful review of the manuscript. We apologize for the error that occurred in the layout of the tables. When the Word version of the manuscript was converted to a PDF file, some tables got displaced. In this revised version of the manuscript, we have rearranged the tables and ensured no such problems exist.

**Figure 6: Panel A says “CCA” – I believe this should be RDA? Or was a canonical correspondence analysis performed?--if so should be in methods near line 261. (note just “CCA” is confusing, because a canonical correlation analysis is a totally different procedure).**

- Response: To address this problem, we have described the method followed to determine CCA and RDA in the place suggested by the reviewer. We added: ‘We have done detrended correspondence analysis (DCA) on the species-sample data (OTU_97_) and chosen the analysis method according to the size of the first axis in the Lengths of gradient part: if the gradient length exceeds 4.0, canonical correspondence analysis (CCA) is preferred; if the gradient is between 3.0–4.0, both RDA and CCA are suitable; if the gradient is shorter than 3.0, RDA is better than CCA." *Lines 261-265*

**Line 473 “adapt” should probably be “****tolerate” – although evolution may sometimes be involved.**

- Response: We agree with the reviewer's suggestion and have made revisions to some inappropriate statements. *Line 476*

**Line 481: “karyotype distribution” should probably be “****community composition”**

- Response: We agree with the reviewer's suggestion and have replaced the inappropriate phrase into“community composition”. *Line 484*

**Line 532: “degradable” should be “able to degrade”**

- Response: We agree with the reviewer's suggestion and have made revisions to some inappropriate statements. *Lines 534-535*

**Thanks again for the reviewer’s help to improve the manuscript.**
